# The laterality of the gallop gait in Thoroughbred racehorses

**DOI:** 10.1371/journal.pone.0198545

**Published:** 2018-06-08

**Authors:** Paulette Cully, Brian Nielsen, Bryony Lancaster, Jessica Martin, Paul McGreevy

**Affiliations:** 1 The Royal (Dick) School of Veterinary Studies and The Roslin Institute, Easter Bush Campus, The University of Edinburgh, Midlothian, United Kingdom; 2 Department of Animal Science, Michigan State University, East Lansing, Michigan, United States of America; 3 Sydney School of Veterinary Science, Faculty of Science, The University of Sydney, Sydney, New South Wales, Australia; Institute of Animal Science, CZECH REPUBLIC

## Abstract

Laterality can be observed as side biases in locomotory behaviour which, in the horse, manifest *inter alia* as forelimb preferences, most notably in the gallop. The current study investigated possible leading-leg preferences at the population and individual level in Thoroughbred racehorses (n = 2095) making halt-to-gallop transitions. Videos of flat races in the UK (n = 350) were studied to record, for each horse, the lead-leg preference of the initial stride into gallop from the starting stalls. Races from clockwise (C) and anti-clockwise (AC) tracks were chosen alternately at random to ensure equal representation. Course direction, horse age and sex, position relative to the inside rail and finishing position were also noted. On C courses, the left/right ratio was 1.15, which represents a significant bias to the left (z = –2.29, p = 0.022), while on AC courses it was 0.92 (z = 0.51, p = 0.610). In both course directions, there was no significant difference between winning horses that led with the left leading leg versus the right (C courses, z = –1.32, p = 0.19 and AC courses, z = –0.74, p = 0.46). Of the 2,095 horses studied 51.26% led with their L fore and 48.74% with their R, with no statistically significant difference (z = -1.16, p = 0.25). Therefore, there was no evidence of a population level motor laterality. Additionally, 22 male and 22 female horses were randomly chosen for repeated measures of leading leg preference. A laterality index was calculated for each of the 44 horses studied using the repeated measures: 22 exhibited right laterality (of which two were statistically significant) and 21 exhibited left laterality (eight being statistically significant); one horse was ambilateral. Using these data, left lateralized horses were more strongly lateralized on an individual level than the right lateralized horses (t = 2.28, p = 0.03, DF = 34) and mares were more left lateralized than males (t = 2.4, p = 0.03, DF = 19).

## Introduction

Laterality refers to the structural and functional differences between the left (L) and right (R) sides of the brain or the body. The physical manifestation of brain lateralisation is observed as variances in perception of stimuli offered to the L or R side of the body, sidedness (or handedness) of motor behaviour [1], and information processing [2].

Laterality is displayed at population or individual levels. Population level laterality exists when most of the individuals (i.e. >50%) in a population have the same directional bias. Population motor lateralities (also referred to as handedness) ranging from 65% up to 90% (in humans) have been reported in many vertebrate species [2]. For individual-level laterality, most individuals of a population exhibit laterality, but the numbers of left- and right-biased individuals are similar so there is no population bias [3].

Anecdotally, equestrian authors have described the motor laterality of horses as a one-sided stiffness to the right [4,5,6] or to the left [7,8], a lack of lateral flexion [9], and preferring a specific leading leg (LL) during the canter [10]. However, it is worth noting that motor laterality may be confused with morphological asymmetry. The advent of equitation science has seen a proliferation of scientific investigations of motor laterality in wild, feral and domesticated equids. Different horse breeds are reported to have variable individual and population handedness [11]. There are also differences in and between populations due to age [12], sex [13,14], training, handling, breeding [15], arousal [16,17], and morphological proportions [18].

In competition horses, motor laterality may affect athletic performance by influencing speed or turning agility and, when evaluating a horse’s potential, understanding motor laterality may inform suitability for a specific sport [19]. Depending on the direction in which they are being raced, individual motor laterality is likely to favour Thoroughbreds that preferentially run with a particular gallop lead (i.e., with a leading leg preference that suits the direction of travel rather than *vice versa*). McGreevy and Thomson [11] found that motor bias became stronger with age and McGreevy and Rogers [15] noted that Thoroughbreds older than two years were significantly more laterally biased than those under two years old. Both age groups contained more L-leg than R-leg-preferent horses.

When completing trials of varying tasks, male horses (n = 20) have been found to exhibit significantly more L leading leg preferences, while female horses (n = 20) exhibited significantly more R leading leg preferences [13]. Similarly, Murphy and Arkins [14] (2008) demonstrated associations between the direction of facial hair whorls and the laterality scores of L-lateralised, R-lateralised and well-balanced horses for performing the motor tasks of walking, cantering and jumping. In contrast, Williams and Norris [20] found no difference in motor laterality between the sexes.

A link between nervousness and motor laterality has been established [21] and breeders may have influenced reactivity in horses that affects what is labelled nervousness. For example, racehorses are bred to be reactive to stimuli, but riding-school horses are preferentially chosen for low reactivity (for traffic safety etc.) [11]. Therefore, understanding, motor bias and associated hemispheric dominance may be useful when forecasting reactive or proactive behaviour [22] and cautiousness [23] as they may affect the type of activity for which a horse is more appropriate [11]. When testing for variances of motor laterality among different breeds of equines, observations of horses standing with all feet on the ground have revealed that standing with one foreleg advanced is the most common indication of motor laterality [11]. These tests have shown that Thoroughbreds were more left lateralised individually than Standardbreds, with no preference found among Quarter Horses [11]. In contrast, Williams and Norris [20] observed a surprisingly strong right leading leg population bias with 90% R and 10% L lateralised animals among Thoroughbreds, Arabians and Quarter Horses in halt-to-gallop transitions. Since racetracks in the USA are all AC, a further investigation into the effects of racing horses on both AC and C tracks on motor laterality is merited, since the direction of the North American tracks may be associated with the preference of gallop leading leg.

No studies to date have investigated the influence of innate motor laterality on the horse’s success in competition, but some authors have proposed connections between kinematic lateralities and compromised performance [24,18]. Musculoskeletal characteristics (even those desired by breeders) are important in horses because they may indicate and influence performance capability [25], injury vulnerability, and disrupt or influence innate movement preferences [18]. Some degree of asymmetry is considered normal [26] but equine skeletal asymmetries have high incidence rates [27] and are linked to laterality [28]. Sizable anatomical asymmetries can affect equine locomotion performance [24], which, beyond a certain point, can constitute lameness [29].

The conformation traits of a small head and long legs that are valued in Thoroughbreds are thought to contribute to lateralised grazing behaviour. This was observed in 46% of 27-week-old Warmblood foals that showed significant laterality when feeding from the ground; a behavioural attribute thought to cause uneven feet, compromise performance and jeopardise soundness [18]. In the study that followed these foals as they matured (until three years old), grazing behaviour and uneven feet were strongly related to sidedness (in trot) [18].

Thoroughbreds have been shown to have longer third metacarpal bones (76% of n = 43) on the right than the left, which could, theoretically, imbue mechanical advantages when racing on AC courses and disadvantages on C courses [30]. In addition, training and competition can affect structural asymmetry. Of 500 Standardbred trotters training and competing on AC tracks, L and R tuber sacrale height differences occurred in 8% of horses (30 lower to the L and 9 lower to the R) with no difference in proportions due to sex. A tuber sacrale height difference of ≥1 cm (either side), perhaps due to asymmetrical load distribution on under-banked curves, was associated with significantly lower earnings, fewer races per horse, working-at-speed difficulties and lower race speed records [24].

Internationally, racehorses compete on C and AC tracks. It has been proposed [11, 15] that horses preferring the right foreleg lead in gallop should perform better on C courses than on AC courses and *vice versa*. The transverse gallop, used most commonly by racing horses, consists of a stance and suspension phase, although a rotary or disunited gallop can also be used by horses where the first and second footfalls are equivalent to the transverse gallop, followed by the forefeet in reverse order [31].

Studies have observed the rotary gallop to occur at the start of a race. Kai and Kubo [32] (1993) observed only three horses galloping once each, two of which exited the starting stalls in a rotary gallop. Hiraga et al. [33] (1994) observed only one horse completing two race starts. Leach and Dag [34] (1983) refer to unpublished data stating that most racehorses accelerate from the standing position in rotary gallop and change to the transverse gallop after a few strides. Also, Rooney [35] (1989) states that horses use the rotary gallop as they exit the starting gates, but these comments are based on personal observation and not on an empirical study. Therefore, in this study, horses are presumed to start races in the transverse gallop. If horses were observed to start from the stalls in a rotary gallop, they were excluded from the observational data.

The aim of the current study was to investigate the existence of motor laterality in Thoroughbreds at the individual and population level, by investigating their gallop leading leg preference on both C and AC courses in the UK. As the first study to investigate the association between the motor laterality of horses and competition success, it was designed to explore how gallop leading leg preferences may be associated with winning races on the flat, as well as with a horse’s sex and age. Our hypotheses (H1-H10) explore various potential influences on observed laterality of horses as they exited the starting stalls. Firstly, four null hypotheses were formulated, as follows: H1. *that racehorses will not have a population preference for their leading leg when exiting the starting stalls in gallop; H2*. *that on C racecourses*, *when racehorses exit the starting stalls in gallop there will be no difference between the number of horses selecting their L leading leg and those horses choosing their R leading leg; H3*. *that on AC racecourses*, *when racehorses exit the starting stalls in gallop there will be no difference between the number of horses selecting their L leading leg and those horses choosing their R leading leg; and H4*. *that racehorses will not have an individual preference for their leading leg when exiting the starting stalls in gallop*. The study was also designed to investigate any differences in leading leg preference associated with sex and age. For this part of the investigation, two null hypotheses were formulated, as follows: H5. *That the leading leg chosen by racehorses when exiting the starting stalls in gallop does not depend on the sex of the horse; and H6*. *that the leading leg chosen by racehorses when exiting the starting stalls in gallop does not depend on the age of the horse*. Also, because visual stimuli may inform the horse as to the direction of course and hence cue the selection of an appropriate leading leg, the association between the position of the starting stall relative to the inside rail and the leading leg selected was examined. For this part of the investigation, two null hypotheses were formulated, as follows: H7. *that the leading leg chosen by racehorses when exiting the starting stalls in gallop on C courses does not depend on the proximity to the inside fence; and H8*. *that the leading leg adopted by racehorses when exiting the starting stalls in gallop on AC courses does not depend on the proximity to the inside fence*. Finally, as the first study to investigate the association between the motor laterality of Thoroughbreds and performance, the study was designed to explore how gallop leading leg preference may be associated with winning. For this part of the investigation, two null hypotheses were formulated, as follows: H9. *that on C racecourses*, *there will be no difference in the number of winning horses between those who adopt a R leading leg and those adopting a L leading leg when exiting the starting stalls in gallop; and H10*. *that on AC racecourses*, *there will be no difference in the number of winning horses between those who select their R leading leg and those which select their L leading leg when exiting the starting stalls in gallop*.

## Method

### Sample size requirements

Minitab was used to calculate the sample size requirements, *a priori*, for the pooled C and AC race starts (using sample size for one proportion). This returned a requirement of 1225 observations having an 80% power to detect with a 95% confidence interval (CI) a statistically significant proportion of L to R or R to L gallop leading leg starts of 54/46%. Given that population proportions of between 65–90% are reported in invertebrates [2], 1225 observations or above were considered sufficient to address the question of population laterality. For detailed observations for individual laterality, Minitab used *a priori* to calculate the sample size requirements for repeated measures indicated that a minimum of 28 individual horses were required at α = 0.5, power = 0.8 and 95% CI levels.

### Racehorse study population

The number of racehorses registered as “in training” for flat races in the UK during the study period (1/10/14 to 30/9/2015) averaged 14,322 per month [36].

### Racecourses and races included in the study

The BRA [35] (2015) provided Excel spreadsheets of planned flat races for 2014 and 2015, which were collated for analysis. The study period from 1/10/14 to 30/9/2015 covered 6257 races. Each race corresponded to a numbered row on the spreadsheet and appropriate random numbers were generated. During the study period, there were n = 22 AC courses and n = 14 C courses operating (see Table 1). Horse numbers competing per race ranged from 2 to 15 and race lengths ranged from 5 furlongs (F, 1100 yards, approximately 1006 meters) to 21 F (129 yards, 4620 yards, approximately 4335 meters).

**Table 1:** UK racecourses operating flat races and the number of races run at each course during the study period 1/10/14 to 30/9/2015 [35]. (BRA, 2015). A total of 3803 races were run over anti-clockwise courses and 2,455 races over clockwise courses. Most track surfaces were turf, but there were also four all-weather (AW) tracks.

ANTI-CLOCKWISE COURSES				CLOCKWISE COURSES			
Course name	Number of races	Course name	Number of races	Course name	Number of races	Course name	Number of races
Ayr	128	Newbury	129	Ascot	114	Salisbury	114
Bath	148	Newcastle	134	Beverley	138	Sandown Park	103
Brighton	153	Nottingham	170	Carlisle	93	Windsor	192
Catterick Bridge	122	Pontefract	111	Chelmsford City	259		
Chepstow	117	Redcar	136	Goodwood	131		
Chester	104	Southwell (AW)	259	Hamilton Park	126		
Doncaster	176	Thirsk	118	Kempton (AW)	528		
Epson Downs	70	Wetherby	30	Leicester	151		
Ffos Las	49	Wolverhampton (AW)	747	Musselburgh	118		
Haydock Park	160	Yarmouth	28	Newmarket	281		
Lingfield Park (AW)	599	York	115	Ripon	107		

### Observational methodology

Streamed head-on videos of horses emerging from starting stalls at the start of flat races (available at http://www.skysports.com/racing/results) were used to observe each horse’s initial gallop leading leg.

Data from AC and C courses were collected alternately to ensure equal representation, and the videos were displayed at high screen resolution (1920 × 1080 pixels) in a darkened room to minimise reflections. If required, the motion was slowed down using a tablet equipped with slow-motion photography and playback on a 4K-resolution screen.

Races were excluded if stalls were not used at the start of the race. Races were also excluded if the front of the stalls was obscured by the camera angle, mist, rain, fog, bumps in the course or the angle of the sun casting shadows, etc. Individual horses were excluded if their forelimb movements were not clearly visible. The shorter races are generally run on the straight, so races that did not include a bend were excluded from the current study. This process resulted in 350 observed races (175 races on C courses and 175 races on AC courses) with the minimum requirement of 1225 individual observations being surpassed. After duplicated observations of individual horses were removed, this yielded data on 1131 horses on C courses and 1113 on AC courses. For C and AC courses combined, once any duplicates had been removed, the total number of horses observed was 2095.

When racing, horses begin from a standing position and break into a gallop when exiting the stalls. The gallop is a transverse, four-beat gait where forelimb and hindlimb footfalls occur in couplets. The leading leg refers to the foreleg that touches the ground after and in advance of the trailing foreleg (TrF). When leading with the R leading foreleg (LF), the sequential order of the limb-to-ground contact is: L trailing hindleg (L TrH), R leading hindleg (LH), L TrF and R LF. When leading with the L fore, the opposite occurs. There is also a stance and suspension phase to the gait. The stance phase (when on the R fore lead), begins with the L TrH contacting the ground and ends when the R LF leaves the ground. When there is no ground contact, this is termed the suspension phase [31].

One observer noted the stride for each horse, the researcher noted the first leg raised as the horse’s forequarters rose to leave the starting stalls. If the L foreleg lifted first, then the stride was recorded as R, denoting a R gallop leading leg and *vice versa*. If the first leg raised was unclear, the last hoof touching the ground was taken to indicate the stride.

For the current study, only horses starting races in the transverse gallop were of interest. The name of each horse observed was noted solely for the purpose of removing duplicates and as a database for random selection of individual animals for repeated measures. Also, the horse’s starting position relative to the inside rail was noted to explore the possibility that visual cues as to course direction might affect leading-leg preference. Finishing position, sex and age were also noted from the online reports of each race (available at http://www.skysports.com/racing/results). Data were anonymised and no horse was singled-out in any way (see S1 File).

For detailed observations for individual laterality using repeated measures, 44 focal horses (11 geldings, 22 mares, and 11 stallions) were randomly chosen from those already observed. Observations from all their available lifetime race videos were taken using the method established above. This yielded between 5 and 46 observations per horse.

### Measuring internal consistency of observations

Testing for observational reproducibility, 50 horses previously scored as L or R were randomly chosen and three further observers were enlisted to separately re-score their leading-leg preference in one race-start, using the method above. Cronbach’s Alpha (CA) was used as a measure of internal consistency among the four sets of observations as it signposts how reliably multiple observers assess the same skill. A low CA result indicates that the method may not be reliable. A typical value of 0.7 or higher is commonly used to indicate an acceptable level of reproducibility [37], so this was set as the target level of consistency for this study.

### Statistical analysis

Minitab was used to calculate the following statistical analyses: the alpha level (α) was set at 0.05, the power at 0.8 and the CI set at 95%. To test for any population laterality among the proportions of L- and R-scored horses on pooled data (H1), the one-proportion z test (using the normal approximation) was employed. To test for any leading-leg differences between the proportions of L- and R-scored horses, on C (H2) and AC (H3) courses individually, the one-proportion z-test was used (again using the normal approximation).

A Binary Logistic Regression (BLR) model was run for AC and C courses jointly to examine any association between the direction of course and the selection of gallop leading leg (H4).

For each subgroup, stallions, geldings, mares, and male and female, a one-proportion test was performed to assess for any statistically significant differences due to sex, between horses that were L- and R-handed (H5). Also, to test for any association between laterality and sex, two subgroups of data were created: 1) males and females; and 2) mares, geldings and stallions, along with their leading-leg observations. A BLR model for each set was run. A BLR model was also run to test whether the preferred gallop leading leg altered with the age of the horse (H6). Each age from 2 to 10 years was entered individually (i.e. not grouped into age ranges). Horses in the 12- and 13-year categories had to be omitted from the calculation because there were too few samples to run the BLR. To explore any association between a horse’s starting position (i.e. proximity to the inside rail, with position 1 being nearest) and gallop leading leg preference, a BLR model was performed for C (H7) and AC (H8) courses separately. To ascertain if there was a significant difference between the proportion of L and R winners on C and AC courses, a one-proportion z-test was conducted. A BLR model was also run for C (H9) and AC (H10) courses jointly to investigate any association between the direction of the course and the leading leg of winning horses.

Testing for individual laterality, a laterality index (LI) [38,39] was calculated for each of the 44 focal horses for which repeated observations were taken. The formula was:

$$LI=\frac{(total\;R-total\;L)\;\times 100}{\left(total\;R+total\;L\right)}$$

If total R observations were equal to total L observations, the LI = 0. A positive index indicated a rightward bias (+100 represents 100% R starts) and, a negative index denoted a leftward bias (–100 represents 100% L starts).

To determine if each of the 44 focal horses were significantly lateralised, the numbers of L- and R-leading leg starts for each horse were used in the one-proportion z-test (using either the normal approximation or exact test, where observation numbers were low). Comparisons among horses found to be significantly L or R lateralised were conducted using a Welch’s t-test to reveal if the degree of laterality was greater in either one or the other. Similar tests were carried out for individuals in all subgroups: all horses, female, male, male and female L leading leg biased, and male and female R leading leg biased.

Histograms (see S1 File) were generated for the male and female, and mares, geldings and stallions subgroups to observe for the presence of a bimodal distribution (a clear set of both L and R lateralised horses), which is indicative of individual, but not population, laterality [40]. To assess if the distribution suggested by the histograms was bimodal, a test to differentiate between unimodal and bimodal populations was employed (see [41], p. 225 for precise method). These involved calculations using the standard deviations (σ) and means (μ) generated within the histograms. The method calculated σ_1/_σ_2_, whose value was used to look up a Mixture Density (MD) value from a table supplied by Schilling et al. [41] (2002). If MD × (σ_1_ + σ_2_) < (μ_1_ – μ_2_), the distribution was bimodal.

Where a bimodal distribution emerged, a test for Equal Variances was run for each of the five groups to test for significance in variances or standard deviations between the L- and R-lateralised horses in each group (using the multiple comparisons p-value). If there were fewer than 20 observations per group, then the lesser value of the multiple comparisons p-value or the Levine’s test p-value was used. A Welch’s t-test was also conducted on the five groups. Where the Equal Variance test indicated either equal variance or no equal variance, the “assume equal variances” box in Minitab during calculations was ticked or unticked accordingly.

Additional testing for any population laterality using the LIs for all 44 horses was carried out using the one-sample t-test. As the t-test requires normally distributed data, an Anderson-Darling Normality Test was run. If the data were not normally distributed, they were transformed using the Johnson Transformation function in Minitab.

A Regression Analysis (RA) testing for laterality, within male and female and within gelding, stallion and mare groups was carried out. The untransformed LI data were used because regression analysis does not require the data to be normally distributed.

## Results

When 50 observations of the gallop leading leg of horses breaking from the starting stalls were repeated by three observers similarly trained to observe the horse’s movements and under the same observing conditions, the Cronbach’s Alpha was calculated at 0.8977.

No horses were observed to start from the stalls in a rotary gallop. There were more L leading leg starts (n = 1074, 52.26%) than R leading leg starts (n = 1021, 48.74%) (pooled data from both C and AC courses), however, this difference was not significant (z = –1.16, CI (0.4659, 0.5087) and p = 0.25). Therefore, there was no evidence of a population level motor laterality.

For C courses, there were more L leading leg starts (n = 604) in gallop than R leading leg (n = 527) starts. The ratio of L to R gallop leading leg starts was 1.15:1.00 (53.51%:46.49%). After performing a one-sample z-test for proportions (z = –2.29, CI (0.4355, 0.4942), p = 0.02), the null hypothesis was rejected and the alternative hypothesis was accepted, concluding there is a significant difference in proportions between L and R gallop leading leg starts on C racecourses.

In contrast, there was no significant difference between the number of horses starting on their L or R gallop leading leg on AC courses (z = 0.51, CI (0.4782, 0.5370), p = 0.61).

Results of the BLR showed that direction of course was significantly associated with the gallop leading leg when exiting the starting stalls (Chi-square = 3.90, degrees of freedom (DF) = 1, p = 0.048). The positive coefficient for C courses (Coef = 0.1669) indicated that C courses were more likely to predict the gallop leading leg of horses exiting the starting stalls than AC courses (with odds of 1.187%).

A one-proportion z-test (z = –1.32, CI (0.377551, 0.51286), p = 0.185) revealed no significant difference between winning horses starting on their L gallop leading leg and those starting on their R gallop leading leg.

There were more L gallop leading leg winners (n = 78, 53.06%) than R gallop leading leg winners (n = 69, 46.94%) on AC courses. However, a one-proportion z-test (z = –0.74, CI (0.4016, 0.5370), p = 0.46) showed no significant difference between the number of winners starting on their L gallop leading leg and those starting on their R gallop leading leg on AC courses.

The BLR model showed that neither C nor AC course direction was statistically significantly associated with the gallop leading leg of winning horses (Chi-square = 0.17, DF = 1, p = 0.68). The starting position relative to the inside fence on C courses did not significantly predict the horse’s leading leg (Chi-square = 6.09, DF = 12, p = 0.911). Furthermore, there was no significant relationship between the starting position relative to the inside fence on AC courses and the horse’s gallop leading leg (Chi-square = 8.40, DF = 12, p = 0.75). Similarly, the age of the horse did not statistically significantly predict the horse’s leading leg (Chi-square = 4.69, DF = 9, p = 0.860).

The BLR model showed no relationship between the sex of the horse and the horse’s gallop leading leg (Chi-square = 1.12, DF = 1, p = 0.29). A further BLR model was run for stallion, gelding and mare subgroups, and no association was found between stallions, geldings or mares with preference of gallop leading leg (Chi-square = 1.54, DF = 2, p = 0.46).

Testing for individual laterality for each of the 44 horses, a laterality index (LI) was calculated and a one-proportion z-test for each horse was performed to understand if horses were individually lateralised. The results for individual horses appear in Table 2.

**Table 2:** Repeated measures of the gallop LL preference of individual horses as they exited the starting stalls and the results of individual statistical analyses (test for one proportion).

HORSE1	SEX2	REPEATED OBSERVATIONS OF GALLOP LL (R or L)3	LI4	Z-VALUE5	P-VALUE6	STATISTICALLY SIGFICANT = S (L or R)7
A	G	RRRRLRRRRRLRLRRRRRLRRLRLRRLL	42.86	2.27	0.023	S(R)
B	G	RRRRLRLRRRLRRRRRRL	55.56	2.36	0.031	S(R)
C	G	LRLLLLLLLLLRLRLLLRLLL	-61.91	-2.84	0.007	S(L)
D	G	LRRRRRRLLLLLRR	14.29	0.53	0.593	
E	G	RRRLLRRR	50.00	1.14	0.289	
F	G	LLLLLLL	-100.00	-2.65	0.016	S(L)
G	G	LLLLLLR	-71.43	-1.89	0.125	
H	G	RLLRRRRRR	55.56	1.67	0.180	
I	G	LLRLLRLLLRLLLLRLR	-41.18	-1.70	0.090	
J	G	LLRLLLLLLLLLRLRL	-62.50	-2.50	0.021	S(L)
K	G	LRRLRLLL	-25.00	-0.71	0.717	
L	M	LLLLRLLLLRRLLLLLRLRRRLLLRLLRRLL	-35.48	-1.98	0.048	S(L)
M	M	RRRLRRRLLRLL	16.67	0.58	0.564	
N	M	RLRRLLRRLLLLRRLRRRLL	0.00	0.00	1.000	
0	M	RLLLLLLLLLLR	-66.67	-2.31	0.039	S(L)
P	M	RRLRRLRRRLRRLR	42.86	1.60	0.180	
Q	M	LLRLRLRLLLRLLLLRLRRRLRLRRL	-15.38	-0.78	0.433	
R	M	RRRLRRRLLLLLRLLL	-12.50	-0.50	0.617	
S	M	RLRRRLLRRL	20.00	0.63	0.754	
T	M	RLLRLLRLRLRRRRLRR	17.65	0.73	0.467	
U	M	LLLLLL	-100.00	-2.45	0.031	S(L)
V	M	LLLRLRRLRRRRL	7.69	0.28	0.782	
W	M	RLRRRRRRLRR	63.64	2.11	0.065	
X	M	RLLRLRRRRLRRRLRLL	17.65	0.73	0.467	
Y	M	LRRRRLLRRRRLRR	42.86	1.60	0.180	
Z	M	LLLLRL	-66.67	-1.63	0.219	
AA	M	RRRRRLRLLLRLRL	14.29	0.53	0.593	
AB	M	LLRLRLLLR	-33.33	-1.00	0.508	
AC	M	LLLLLLLL	-100.00	-2.83	0.008	S(L)
AD	M	LLLLLLR	-71.43	-1.89	0.125	
AE	M	RRLRLRRRR	55.56	1.67	0.180	
AF	M	LLLLRLLRLL	-60.00	-1.90	0.109	
AG	M	LRLRRLRR	25.00	0.71	0.727	
AH	S	RRLRLRRRLRLRLLLLLLR	-5.26	-0.23	0.819	
AI	S	RLLLLRRRRRRL	16.67	0.58	0.564	
AJ	S	LLRLRRLRLLLR	-16.67	-0.58	0.564	
AK	S	RLLLLL	-66.67	-1.63	0.219	
AL	S	RLRRRLR	42.86	1.13	0.453	
AM	S	LRRLLRRLR	11.11	0.33	1.000	
AN	S	RRLRR	60.00	1.34	0.375	
AO	S	RRLRRRL	42.86	1.13	0.453	
AP	S	LLRLLLLLLRLLLLLLLRLLLLRLRLLRRLLLLLLLLRRRLLRLRL	-48.94	-3.35	0.001	S(L)
AQ	S	RLRRRRLLLLLLLLLLLRLLR	-33.33	-1.53	0.127	
AR	S	LRRLRRRRR	55.56	1.67	0.180	
Median LI for L preferent horses			-60.00			
Median LI for R preferent horses			42.86			

Anonymized horses are presented in Column 1. Column 2 identifies the sex of the horse: G-gelding, M = mare and S = stallion. In Column 3, the L and R LL observations for each horse appear in chronological order; oldest to the most recent (from L to R).

Individually, 43/44 horses exhibited some degree of laterality and 1 horse was ambilateral, having equal numbers of L and R gallop leading leg starts. Some horses (n = 22) exhibited a trend towards R laterality and 2 of them were significantly lateralised (p≤0.05); 21 horses exhibited a L laterality trend with 8 of them being significantly lateralised (p≤0.05). Of the significantly lateralised horses, there were 2 R and 2 L geldings, 4 L females and 1 L stallion.

The distributions of all horses combined, females, male, geldings, and stallions were found to be bimodal (see Table 3). The presence of a bimodal distribution (a clear set of both L and R lateralised horses) is indicative of individual, but not population, laterality.

**Table 3:** Calculations for the bimodality test designed by Schilling et al. [41] (2002) demonstrate that the groups (all horses, females, males, geldings, and stallions) are all bimodal distributions.

Group	MD = σ_1_/σ_2_	σ_1_+σ_2_	MD×(σ_1_+σ_2_)	μ_1_–μ_2_	Is MD×(σ_1_+σ_2_) < (μ_1_–μ_2_)?
*All horses*	0.65	47.76	55.40	87.16	Yes
*Female*	0.59	49.93	56.42	85.59	Yes
*Male*	0.67	27.57	53.01	89.10	Yes
*Geldings*	0.67	25.76	49.86	103.98	Yes
*Stallions*	0.86	24.58	49.58	72.34	Yes

Results of the Welch’s t-test performed on the various groups are presented in Table 4.

**Table 4:** Results of the Welch’s t-test between the degree of motor laterality to the L and R in groups of significantly lateralised horses (all horses, females, males, geldings, stallions, females L vs males L, and females R vs males R). Significant results are highlighted in bold. The values were calculated using μL-μR = 0 in Minitab (DF = degrees of freedom).

Horses under comparison	t value	p value	DF
*Significantly L lateralised horses vs significantly R lateralised horses*	1.21	0.26	8
*All horses L bias vs all horses R bias*	2.28	**0.029**	34
*Females tending to L bias vs females tending to R bias*	2.40	**0.027**	19
*Males tending to L bias vs males tending to R bias*	0.78	0.44	20
*Geldings tending to L bias vs geldings tending to R bias*	1.23	0.25	9
*Stallions tending to L bias vs stallions tending to R bias*	–0.30	0.77	9
*Females tending to L bias vs males tending to L bias*	0.60	0.57	19
*Females tending to R bias vs males tending to R bias*	–1.43	0.17	20

To further test for individual laterality associated with the sex of the horse, an RA was performed using the repeated measures data on two sub-groups: 1. male/female, and 2. geldings/stallions/mares (entered as categorical data). The leading leg preference was entered as the response variable. For both groups, there was no significant association of sex with preference of leading leg (F = O.21, DF = 1, p = 0.65 and F = 0.47, DF = 2, p = 0.63 respectively), further ruling-out an association between the two.

The data on LIs of the 44 focal horses were not normally distributed and so had to be transformed using the Johnson Transformation. The subsequent t-test found no evidence of population laterality (t = 0.32, p = 0.75).

## Discussion

This study found that for the motor laterality indicator of halt-to-gallop, horses exhibited individual laterality, but no population laterality was found. We acknowledge that lateralised behaviour can vary due to the motor task performed [42]. Grzimek [43] noted this when observing the preferred leading leg horses used when performing several tasks including galloping riderless, pawing the ground and transitioning from halt to walk. Only two of these tasks, pawing and starting to walk, were aligned, with more significantly R lateralised horses than L horses. In contrast, for galloping riderless, there were more significantly L lateralised horses than R horses, demonstrating that each task may not be relying on the same process and that motor behaviour may be task dependent.

The current results align with Wishaw [44], who found no evidence of population laterality and only a few horses that were individually lateralised. Duel and Lawrence [45] noted that ridden horses were more likely to initiate gallop with the L leading leg, but their study (n = 4) was too small to assess for population asymmetry. The absence of evidence of population laterality appears to contradict Vallortigara and Rogers’ [2] prey-predator model for the emergence of motor laterality, instead aligning with the notion that quadrupedal movement impedes the expression of laterality [46].

Repeated measures observations in a focal group of 44 horses in the current cohort showed varying degrees of individual motor laterality ranging from significantly L lateralised (18% of the 44) to significantly R lateralised (4.5%). Individual horses that were left lateralized were more strongly lateralized than individual horses that were lateralized to the right.

Grzimek [43] found remarkably similar percentages of significantly lateralised animals (16% L leading leg and 7% R leading leg) for horses galloping riderless. This alignment implies that, in the current study, jockeys did not significantly influence the initial gallop leading leg preference of horses. However, our findings run counter to those of Williams and Norris [20], who found that Thoroughbreds, Arabians (racing on AC tracks) and Quarter Horses (racing on straight tracks) exhibited a R population laterality of 90%. Such a strong motor laterality has previously been reported only in humans and in parrots (*Psittaciformes*) which preferentially use the L foot [47]. Because the Williams and Norris [20] study and the current study were similar in methodology, it is important to look more closely at what may have caused the results to differ so markedly.

Thoroughbreds in the UK are usually trained on straight tracks at home, whereas in the US horses are largely trained at the tracks on which they race (and which are only in AC direction) [48]. In the US assistant starters are employed to keep horses under control in the starting stalls. The assistant starter stands on a ledge inside the starting stall to the left of the horse, maintaining control via a lead rope attached to the bridle. The assistant starter can pull the horse’s head and neck to the left and can, if the horse becomes fractious, use harsher methods, such as seizing the ear or pulling the tail laterally or dorsally [49]. This could disrupt the horse’s balance [50] or position the horse’s right shoulder such that it is perhaps encouraged to initiate gallop on the R leading leg. Also, the horse may be motivated to escape from the assistant starter (on its left), which again might prompt such horses to favour the R leading leg start.

On C courses only, there was a significant bias to start on the L lead. We cannot rule-out pain as a contributor to the current findings. There seems no plausible explanation for different leading leg preferences on C and AC courses. Given that, over the study period, there were more races in the AC direction (n = 3,802) than in the C direction (n = 2,455) (see Table 1), perhaps experienced horses become accustomed to running to the L. This might account for the significant numbers starting on the L lead on C courses but not for the almost equal numbers of horses starting on either the L or R lead on AC courses. Therefore, this finding merits further investigation.

Turning to the effect of age on motor laterality, the current results show no evidence of changing motor laterality with age. However, this does not discount the possibility that age-related changes may have occurred prior to the age of two (the age of the youngest horses in the study). Wishaw’s [43] study observed only 3-year-old horses, while Williams and Norris [20] and Grzimek [46] did not consider age.

The present study found no evidence of any sex differences in direction of laterality, but the degree of motor laterality was significantly stronger to the L in females, though not in males. This may suggest that this phenomenon is more strongly innate in female horses or that the effect of handling on females is different from that on males. Williams and Norris [20] (2007) found no difference in handedness between the sexes and the other comparison studies did not test for this. To the authors’ knowledge, no studies have investigated any association between observed motor laterality of horses and competition success (winning). In the current study, there was no significant difference between winning horses that preferred to start on their L or R leading leg in either course direction.

From the repeated measures data, the results have shown that at least 22.7% of horses (total percentage of horses that were significantly lateralised to the L and to the R), may be at an elevated risk of injury if raced in a direction contra to their preferred leading leg. On bends, it is common for horses to use the L leading leg when galloping in an AC direction and vice versa [51]. At racing speed, vertical ground reaction forces (VGRF) on banked bends are greatest in the LF, followed by the TrF, LH and TrH [52]. On the straight, the highest to lowest VGRF sequence is: LF, LH, TrF and TrH [53]. In conjunction with this, racecourse injuries associated with the leading leg at the time of accident have been recorded. Most injuries (72%) occur to the leading leg (irrespective of course direction). Major sites of injury are on the straights (where 30.77% of injuries occurring here involve the leading leg); passing a turn (where 62.5% of injuries occurring here correspond with the leading leg); and coming out of a turn (where 55.31% of injuries are to the leading leg) [54]. This implies that the greater tendency to fracture the LF is due to the greater strain put on that leg during turns. Also, of injuries sustained to the leading leg, horses are more likely to fracture the foreleg contralateral to the leading leg (i.e. the non-leading leg) used for the first few strides of a race [55]. This would suggest that the non-leading leg side has either less agility or an underlying weakness. Therefore, horses racing on their weaker side, in a direction contra to their preferred leading leg could be at increased risk of injury and wastage. Identifying that a horse is L- or R-handed could allow trainers to develop the weaker side and produce a more balanced or ambilateral horse. Equally, in countries that race in both directions (such as the UK and Australia), it could allow trainers to prepare strongly biased horses preferentially for races in certain directions.

Finally, the current study revealed that, even when the methodology and breed of horse are similar, the same indicator of motor laterality can reveal different population- and individual-level handedness. This is probably due to a suite of unquantifiable outside influences affecting the leading leg preference of horses at the gallop, so it is difficult to make comparisons between this and previous studies. Clearly, comparisons of the current data with investigations conducted on different indicators of motor laterality are even more problematic. A useful future project could develop a universal set of criteria for the continuum between significantly L and significantly R lateralised animals, such that laterality studies could report results that are directly comparable. This could follow the methodology of Fagard et al. [56] (2015).

### Limitations

The race videos available were not always suitable for data collection due to imaging issues such as unsuitable camera angles and raindrops on the camera lens. This meant that the number of observations per horse ranged from 5 to 46 for the repeated-measures aspect of the current study. Ideally, future comparable studies of archived videos of racing horses, should source more observations per horse, if possible. Also, future focal studies of this sort should consider the age of horses in more detail and could reveal changes in the strength of laterality over time.

Although the current methodology is reproducible, as evidenced by the Cronbach’s test, processing such a large sample is time consuming. Not all winning horses could be assessed for laterality because the legs of some were obscured from view as they exited the stalls, which yielded fewer horses than hoped. In 350 races, only 293 winning horses could be evaluated. This may have led to a type II error in finding the higher numbers of L-leading-leg-preferent horses non-significant when the result may have been significant with a higher number of observations.

## Conclusions

The repeatability of observations in this study was high and yielded data on 2095 individual horses. After the visual cue of the inside rail was eliminated as a possible influence on the horse’s leading leg preference, the current results suggest that Thoroughbreds do not display motor laterality at the population level. However, using repeated measures analysis, a sample of the horses was found to exhibit varying degrees of individual motor laterality and the degree of laterality was significantly greater to the L than to the R. This was an attribute of females but not other subgroups. Gallop leading leg preferences did not alter with age, although during the horse’s formative years, any effect on motor laterality due to maturation prior to starting their racing careers cannot be discounted. On C courses, horses were significantly more likely to start on the L leading leg than on the R, but on AC courses there was no significant difference between the number of L and R starts, a phenomenon that calls for further investigation. In addition, there was no significant association between the leading leg selected and winning in either course direction. Furthermore, at least 22.7% of horses studied repeatedly have a significant bias and may at times be racing in a direction contra to what might be considered their innate gallop leading leg preference. It seems that training (or lack of it) is not conditioning horses to become more ambilateral. The role of pain as contributor to motor laterality in the current population cannot be quantified but certainly merits consideration. Given that injury during racing is more likely to occur to the non-leading leg used for the first few strides of a race and the leading leg on turns (in both C and AC directions), the bias reported here may be placing horses and jockeys at risk.

## Supporting information

S1 File Thoroughbred racehorse data. (DOCX)
