# Supplementary material for: The laterality of the gallop gait in Thoroughbred racehorses
Source: PLoS One. 2018 Jun 8;13(6):e0198545. doi: 10.1371/journal.pone.0198545 (PMC5993273; doi:10.1371/journal.pone.0198545)
Supplement: S1 File — (DOCX) [file pone.0198545.s001.docx]

| SUPPLEMENTARY MATERIAL | PAGE |
| --- | --- |
| 1) NOMENCLATURE | 1 |
| 2) Test and CI for One Proportion: RIGHT VS LEFT LEG PREFERENT HORSES ON C AND AC COURSES COMBINED | 1 |
| 3) Test and CI for One Proportion: RIGHT VS LEFT LEG PREFERENT HORSES ON C COURSES | 1 |
| 4) Test and CI for One Proportion: RIGHT VS LEFT LEG PREFERENT HORSES ON AC COURSES | 1 |
| 5) Binary Logistic Regression: LEFT AND RIGHT LEG PREFERENT HORSES versus COURSE DIRECTION | 2 |
| 6) Test and CI for One Proportion: RIGHT AND LEFT LEG PREFERENT WINNERS ON C COURSES | 3 |
| 7) Test and CI for One Proportion: RIGHT AND LEFT LEG PREFERENT WINNERS ON AC COURSES | 3 |
| 8) Binary Logistic Regression: LEFT AND RIGHT LEG PREFERENT WINNERS versus COURSE DIRECTION | 4 |
| 9) Binary Logistic Regression: RIGHT AND LEFT LEG PREFERENT HORSES versus RELATIVE POSITION FROM INSIDE RAIL ON C COURSES | 5 |
| 10) Binary Logistic Regression: LEFT AND RIGHT LEG PREFERENT HORSES versus RELATIVE POSITION FROM INSIDE RAIL ON AC COURSES | 10 |
| 11) Binary Logistic Regression: LEFT AND RIGHT LEG PREFERENT HORSES versus AGE (YEARS) | 15 |
| 12) Test and CI for One Proportion: RIGHT VS LEFT LEG PREFERENT HORSES, STALLIONS | 20 |
| 13) Test and CI for One Proportion: RIGHT VS LEFT LEG PREFERENT HORSES, GELDINGS | 21 |
| 14) Test and CI for One Proportion: RIGHT VS LEFT LEG PREFERENT HORSES, MARES | 21 |
| 15) Test and CI for One Proportion: RIGHT VS LEFT LEG PREFERENT HORSES, MALE | 21 |
| 16) Binary Logistic Regression: LEFT VS RIGHT LEG PREFERENT HORSES versus SEX (MALE OR FEMALE) | 21 |
| (SUPPLEMENTARY MATERIAL CONTINUED) | PAGE |
| 17) Binary Logistic Regression: LEFT AND RIGHT LEG PREFERENT HORSES versus SEX | 23 |
| 18) HISTOGRAMS AND EQUAL VARIANCE TEST RESULTS GENERATED FROM LI DATA All horses   1. All horses 2. Female horses 3. Male horses 4. Geldings 5. Stallions | 25  25  26  27  28  29 |
| 19) FURTHER TEST OF POPULATION LATERALITY USING LI DATA  A) NORMALITY TEST AND JOHNSON TRANSFORMATION OF LI DATA  B) One-Sample T: NORMALISED TRANSFORMED LI DATA | 30  30  30 |
| 20) ONE PROPORTION Z TESTS FOR SIGNIFICANCE OF LATERALITY OF INDIVIDUAL HORSES USING REPEATED MEASURES DATA | 31 |
| 21) WELCH’S T TEST  A) Two-Sample T-Test and CI: LEFT SIGNIFICANTLY LATERALIZED VS RIGHT SIGNIFICANTLY LATERALIZED (ALL HORSES)  B) Two-Sample T-Test and CI: ALL HORSES LEFT VS ALL HORSES RIGHT LEG PREFERENT HORSES  C) Two-Sample T-Test and CI: LI MARES LEFT VS MARES RIGHT LEG PREFERENT  D) Two-Sample T-Test and CI: LI GELDINGS LEFT VS GELDINGS RIGHT LEG PREFERENT  E) Two-Sample T-Test and CI: STALLIONS LEFT VSSTALLIONS RIGHT LEG PREFERENT  F) Two-Sample T-Test and CI: MALE LEFT VS MALERIGHT LEG PREFEERENT  G) Two-Sample T-Test and CI: FEMALE LEFT VS MALE LEFT LEG PREFERENT  H) Two-Sample T-Test and CI: FEMALE RIGHT VS MALE RIGHT LEG PREFERENT | 41  41  41  42  42  42  42  43  43  43 |
| 22) FURTHER TEST OF LATERALITY DUE TO SEX OF THE HORSE USING LI DATA  A) Regression Analysis: LI OF ALL HORSES versus MARES, STALLIONS AND GELDINGS  B) Regression Analysis: LI ALL HORSES versus MALE AND FEMALE HORSES USING LI DATA | 44  44  45 |
| 23) CRONBACH'S ALPHA TEST | 45 |

**1) NOMENCLATURE**

CLOCKWISE (C)

CLOCKWISE (AC)

LATERALITY INDEX (LI)

SIGNIFICANT RESULTS HIGHLIGHTED IN YELLOW

**2) Test and CI for One Proportion: RIGHT VS LEFT LEG PREFERENT HORSES ON C AND AC COURSES COMBINED**

Test of p = 0.5 vs p ≠ 0.5

Event = RIGHT

Variable X N Sample p 95% CI Z-Value P-Value

LEFT/RIGHT ALL COURSES 1021 2095 0.487351 (0.465947, 0.508754) -1.16 0.247

Using the normal approximation.

**3) Test and CI for One Proportion: RIGHT VS LEFT LEG PREFERENT HORSES ON C COURSES**

Test of p = 0.5 vs p ≠ 0.52

Event = RIGHT

Variable X N Sample p 95% CI Z-Value P-Value

LEFT/RIGHT C COURSES 527 1131 0.465959 (0.436887, 0.495032) -2.29 0.022

Using the normal approximation.

4) Test and CI for One Proportion: RIGHT VS LEFT LEG PREFERENT HORSES ON AC COURSES

Test of p = 0.5 vs p ≠ 0.5

Event = RIGHT

Variable X N Sample p 95% CI Z-Value P-value

LEFT/RIGHT A-C COURSES 565 1113 0.507637 (0.478266, 0.537008) 0.51 0.610

Using the normal approximation.

**5) Binary Logistic Regression: LEFT AND RIGHT LEG PREFERENT HORSES versus COURSE DIRECTION**

Method

Link function Logit

Categorical predictor coding (1, 0)

Rows used 2244

Response Information

Variable Value Count

LEFT/RIGHT LEAD LEFT 1152 (Event)

RIGHT 1092

Total 2244

Deviance at Each Iterative Step

Step Deviance

1 3105.590398

2 3105.338557

3 3105.338557

Deviance Table

Source DF Seq Dev Contribution Adj Dev Adj Mean Chi-Square P-Value

Regression 1 3.90 0.13% 3.90 3.902 3.90 0.048

COURSE DIRECTION 1 3.90 0.13% 3.90 3.902 3.90 0.048

Error 2242 3105.34 99.87% 3105.34 1.385

Total 2243 3109.24 100.00%

Model Summary

Deviance Deviance

R-Sq R-Sq(adj) AIC

0.13% 0.09% 3109.34

Coefficients

Term Coef SE Coef 95% CI Z-Value P-Value VIF

Constant -0.0306 0.0600 (-0.1481, 0.0870) -0.51 0.610

COURSE DIRECTION

CLOCKWISE 0.1669 0.0845 ( 0.0012, 0.3326) 1.97 0.048 1.00

Odds Ratios for Categorical Predictors

Level A Level B Odds Ratio 95% CI

COURSE DIRECTION

CLOCKWISE ANTICLOCKWISE 1.1817 (1.0012, 1.3946)

Odds ratio for level A relative to level B

Regression Equation

P(LEFT) = exp(Y')/(1 + exp(Y'))

Y' = -0.0306 + 0.0 COURSE DIRECTION_ANTICLOCKWISE + 0.1669 COURSE DIRECTION_CLOCKWISE

Goodness-of-Fit Tests

Test DF Chi-Square P-Value

Deviance 2242 3105.34 0.000

Pearson 2242 2244.00 0.484

Hosmer-Lemeshow 0 0.00 *

Measures of Association

Pairs Number Percent Summary Measures Value

Concordant 341260 27.1 Somers’ D 0.04

Discordant 288796 23.0 Goodman-Kruskal Gamma 0.08

Ties 627928 49.9 Kendall’s Tau-a 0.02

Total 1257984 100.0

Association is between the response variable and predicted probabilities

**6) Test and CI for One Proportion: RIGHT AND LEFT LEG PREFERENT WINNERS ON C COURSES**

Test of p = 0.5 vs p ≠ 0.5

Event = RIGHT

Variable X N Sample p 90% CI Z-Value P-Value

LEG WINNERS CLOCKWISE 65 146 0.445205 (0.377551, 0.512860) -1.32 0.185

Using the normal approximation.

**7)Test and CI for One Proportion: RIGHT AND LEFT LEAD LEG PREFERENT WINNERS ON AC COURSES**

Test of p = 0.5 vs p ≠ 0.5

Event = RIGHT

Variable X N Sample p 90% CI Z-Value P-Value

WINNERS ANTI CLOCKWISE 69 147 0.469388 (0.401682, 0.537093) -0.74 0.458

Using the normal approximation.

**8) Binary Logistic Regression: LEFT AND RIGHT LEG PREFERENT WINNERS versus COURSE DIRECTION**

Method

Link function Logit

Categorical predictor coding (1, 0)

Rows used 293

Response Information

Variable Value Count

WINNERS ALL COURSES LEFT 159 (Event)

RIGHT 134

Total 293

Deviance at Each Iterative Step

Step Deviance

1 403.978904

2 403.875932

3 403.875931

4 403.875931

Deviance Table

Source DF Seq Dev Contribution Adj Dev Adj Mean Chi-Square P-Value

Regression 1 0.173 0.04% 0.173 0.1726 0.17 0.678

DIRECTION 1 0.173 0.04% 0.173 0.1726 0.17 0.678

Error 291 403.876 99.96% 403.876 1.3879

Total 292 404.049 100.00%

Model Summary

Deviance Deviance

R-Sq R-Sq(adj) AIC

0.04% 0.00% 407.88

Coefficients

Term Coef SE Coef 95% CI Z-Value P-Value VIF

Constant 0.123 0.165 (-0.201, 0.447) 0.74 0.458

DIRECTION

CLOCK 0.097 0.235 (-0.362, 0.557) 0.42 0.678 1.00

Odds Ratios for Categorical Predictors

Level A Level B Odds Ratio 95% CI

DIRECTION

CLOCK ANTI CLOCK 1.1024 (0.6960, 1.7459)

Odds ratio for level A relative to level B

Regression Equation

P(LEFT) = exp(Y')/(1 + exp(Y'))

Y' = 0.123 + 0.0 DIRECTION_ANTI CLOCK + 0.097 DIRECTION_CLOCK

Goodness-of-Fit Tests

Test DF Chi-Square P-Value

Deviance 291 403.88 0.000

Pearson 291 293.00 0.456

Hosmer-Lemeshow 0 0.00 *

Measures of Association

Pairs Number Percent Summary Measures Value

Concordant 5589 26.2 Somers’ D 0.02

Discordant 5070 23.8 Goodman-Kruskal Gamma 0.05

Ties 10647 50.0 Kendall’s Tau-a 0.01

Total 21306 100.0

Association is between the response variable and predicted probabilities

**9) Binary Logistic Regression: RIGHT AND LEFT LEG PREFERENT HORSES versus RELATIVE POSITION FROM INSIDE RAIL ON C COURSES**

Method

Link function Logit

Categorical predictor coding (1, 0)

Rows used 1122

Response Information

Variable Value Count

RIGHT/LEFT LEAD CLOCKWISE COURS LEFT 598 (Event)

RIGHT 524

Total 1122

Deviance at Each Iterative Step

Step Deviance

1 1544.832509

2 1544.447534

3 1544.447493

4 1544.447493

Deviance Table

Source DF Seq Dev Contribution Adj Dev Adj Mean Chi P-Value

Regression 12 6.09 0.39% 6.09 0.507 6.09 0.911

RELATIVE POSITION C COURSES 12 6.09 0.39% 6.09 0.5076 6.09 0.911

Error 1109 1544.45 99.61% 1544.45 1.3926

Total 1121 1550.54 100.00%

Model Summary

Deviance Deviance

R-Sq R-Sq(adj) AIC

0.39% 0.00% 1570.45

Coefficients

Term Coef SE Coef 95% CI Z-Value P-Value VIF

Constant 0.405 0.209 (-0.005, 0.816) 1.94 0.053

RELATIVE POSITION C COURSES

POSITION ELEVEN -0.341 0.416 (-1.156, 0.474) -0.82 0.412 1.30

POSITION FIVE -0.372 0.279 (-0.918, 0.175) -1.33 0.183 2.05

POSITION FOUR -0.179 0.277 (-0.721, 0.364) -0.65 0.518 2.08

POSITION NINE -0.228 0.308 (-0.832, 0.376) -0.74 0.460 1.73

POSITION ONE -0.436 0.272 (-0.970, 0.098) -1.60 0.110 2.16

POSITION SEVEN -0.205 0.290 (-0.774, 0.364) -0.71 0.480 1.90

POSITION SIX -0.405 0.281 (-0.956, 0.145) -1.44 0.149 2.02

POSITION TEN -0.292 0.346 (-0.970, 0.386) -0.84 0.398 1.50

POSITION THIRTEEN 0.693 0.843 (-0.959, 2.345) 0.82 0.411 1.06

POSITION THREE -0.307 0.277 (-0.850, 0.236) -1.11 0.268 2.08

POSITION TWELVE 0.105 0.557 (-0.987, 1.198) 0.19 0.850 1.15

POSITION TWO -0.313 0.273 (-0.849, 0.223) -1.15 0.252 2.14

Odds Ratios for Categorical Predictors

Level A Level B Odds Ratio 95% CI

RELATIVE POSITION C COURSES

POSITION ELEVEN POSITION EIGHT 0.7111 (0.3147, 1.6070)

POSITION FIVE POSITION EIGHT 0.6897 (0.3993, 1.1913)

POSITION FOUR POSITION EIGHT 0.8364 (0.4863, 1.4384)

POSITION NINE POSITION EIGHT 0.7963 (0.4354, 1.4564)

POSITION ONE POSITION EIGHT 0.6468 (0.3793, 1.1030)

POSITION SEVEN POSITION EIGHT 0.8148 (0.4613, 1.4393)

POSITION SIX POSITION EIGHT 0.6667 (0.3844, 1.1563)

POSITION TEN POSITION EIGHT 0.7467 (0.3791, 1.4705)

POSITION THIRTEEN POSITION EIGHT 2.0000 (0.3833, 10.4359)

POSITION THREE POSITION EIGHT 0.7356 (0.4274, 1.2660)

POSITION TWELVE POSITION EIGHT 1.1111 (0.3728, 3.3120)

POSITION TWO POSITION EIGHT 0.7312 (0.4279, 1.2493)

POSITION FIVE POSITION ELEVEN 0.9698 (0.4395, 2.1401)

POSITION FOUR POSITION ELEVEN 1.1761 (0.5346, 2.5876)

POSITION NINE POSITION ELEVEN 1.1198 (0.4873, 2.5732)

POSITION ONE POSITION ELEVEN 0.9095 (0.4158, 1.9895)

POSITION SEVEN POSITION ELEVEN 1.1458 (0.5112, 2.5683)

POSITION SIX POSITION ELEVEN 0.9375 (0.4236, 2.0747)

POSITION TEN POSITION ELEVEN 1.0500 (0.4324, 2.5496)

POSITION THIRTEEN POSITION ELEVEN 2.8125 (0.4895, 16.1601)

POSITION THREE POSITION ELEVEN 1.0345 (0.4700, 2.2770)

POSITION TWELVE POSITION ELEVEN 1.5625 (0.4553, 5.3624)

POSITION TWO POSITION ELEVEN 1.0282 (0.4695, 2.2520)

POSITION FOUR POSITION FIVE 1.2127 (0.7313, 2.0110)

POSITION NINE POSITION FIVE 1.1546 (0.6522, 2.0442)

POSITION ONE POSITION FIVE 0.9378 (0.5707, 1.5411)

POSITION SEVEN POSITION FIVE 1.1815 (0.6924, 2.0159)

POSITION SIX POSITION FIVE 0.9667 (0.5777, 1.6176)

POSITION TEN POSITION FIVE 1.0827 (0.5658, 2.0717)

POSITION THIRTEEN POSITION FIVE 2.9000 (0.5623, 14.9574)

POSITION THREE POSITION FIVE 1.0667 (0.6428, 1.7701)

POSITION TWELVE POSITION FIVE 1.6111 (0.5501, 4.7184)

POSITION TWO POSITION FIVE 1.0602 (0.6439, 1.7457)

POSITION NINE POSITION FOUR 0.9521 (0.5400, 1.6786)

POSITION ONE POSITION FOUR 0.7733 (0.4729, 1.2647)

POSITION SEVEN POSITION FOUR 0.9742 (0.5735, 1.6549)

POSITION SIX POSITION FOUR 0.7971 (0.4786, 1.3277)

POSITION TEN POSITION FOUR 0.8928 (0.4683, 1.7020)

POSITION THIRTEEN POSITION FOUR 2.3913 (0.4643, 12.3159)

POSITION THREE POSITION FOUR 0.8796 (0.5325, 1.4527)

POSITION TWELVE POSITION FOUR 1.3285 (0.4546, 3.8822)

POSITION TWO POSITION FOUR 0.8742 (0.5335, 1.4327)

POSITION ONE POSITION NINE 0.8122 (0.4644, 1.4205)

POSITION SEVEN POSITION NINE 1.0233 (0.5657, 1.8509)

POSITION SIX POSITION NINE 0.8372 (0.4710, 1.4881)

POSITION TEN POSITION NINE 0.9377 (0.4667, 1.8841)

POSITION THIRTEEN POSITION NINE 2.5116 (0.4774, 13.2150)

POSITION THREE POSITION NINE 0.9238 (0.5236, 1.6298)

POSITION TWELVE POSITION NINE 1.3953 (0.4623, 4.2117)

POSITION TWO POSITION NINE 0.9182 (0.5241, 1.6088)

POSITION SEVEN POSITION ONE 1.2598 (0.7481, 2.1216)

POSITION SIX POSITION ONE 1.0308 (0.6244, 1.7015)

POSITION TEN POSITION ONE 1.1545 (0.6098, 2.1855)

POSITION THIRTEEN POSITION ONE 3.0923 (0.6021, 15.8821)

POSITION THREE POSITION ONE 1.1374 (0.6950, 1.8615)

POSITION TWELVE POSITION ONE 1.7179 (0.5904, 4.9989)

POSITION TWO POSITION ONE 1.1305 (0.6963, 1.8355)

POSITION SIX POSITION SEVEN 0.8182 (0.4775, 1.4019)

POSITION TEN POSITION SEVEN 0.9164 (0.4699, 1.7870)

POSITION THIRTEEN POSITION SEVEN 2.4545 (0.4723, 12.7563)

POSITION THREE POSITION SEVEN 0.9028 (0.5311, 1.5347)

POSITION TWELVE POSITION SEVEN 1.3636 (0.4603, 4.0400)

POSITION TWO POSITION SEVEN 0.8974 (0.5318, 1.5141)

POSITION TEN POSITION SIX 1.1200 (0.5833, 2.1506)

POSITION THIRTEEN POSITION SIX 3.0000 (0.5808, 15.4945)

POSITION THREE POSITION SIX 1.1034 (0.6620, 1.8393)

POSITION TWELVE POSITION SIX 1.6667 (0.5679, 4.8914)

POSITION TWO POSITION SIX 1.0968 (0.6631, 1.8141)

POSITION THIRTEEN POSITION TEN 2.6786 (0.4949, 14.4981)

POSITION THREE POSITION TEN 0.9852 (0.5165, 1.8794)

POSITION TWELVE POSITION TEN 1.4881 (0.4727, 4.6849)

POSITION TWO POSITION TEN 0.9793 (0.5165, 1.8567)

POSITION THREE POSITION THIRTEEN 0.3678 (0.0714, 1.8948)

POSITION TWELVE POSITION THIRTEEN 0.5556 (0.0836, 3.6903)

POSITION TWO POSITION THIRTEEN 0.3656 (0.0711, 1.8788)

POSITION TWELVE POSITION THREE 1.5104 (0.5167, 4.4153)

POSITION TWO POSITION THREE 0.9940 (0.6061, 1.6301)

POSITION TWO POSITION TWELVE 0.6581 (0.2259, 1.9167)

Odds ratio for level A relative to level B

Regression Equation

P(LEFT) = exp(Y')/(1 + exp(Y'))

Y' = 0.405 + 0.0 RELATIVE POSITION C COURSES_POSITION EIGHT

- 0.341 RELATIVE POSITION C COURSES_POSITION ELEVEN

- 0.372 RELATIVE POSITION C COURSES_POSITION FIVE

- 0.179 RELATIVE POSITION C COURSES_POSITION FOUR

- 0.228 RELATIVE POSITION C COURSES_POSITION NINE

- 0.436 RELATIVE POSITION C COURSES_POSITION ONE

- 0.205 RELATIVE POSITION C COURSES_POSITION SEVEN

- 0.405 RELATIVE POSITION C COURSES_POSITION SIX

- 0.292 RELATIVE POSITION C COURSES_POSITION TEN

+ 0.693 RELATIVE POSITION C COURSES_POSITION THIRTEEN

- 0.307 RELATIVE POSITION C COURSES_POSITION THREE

+ 0.105 RELATIVE POSITION C COURSES_POSITION TWELVE

- 0.313 RELATIVE POSITION C COURSES_POSITION TWO

Goodness-of-Fit Tests

Test DF Chi-Square P-Value

Deviance 1109 1544.45 0.000

Pearson 1109 1122.00 0.386

Hosmer-Lemeshow 6 0.00 1.000

Measures of Association

Pairs Number Percent Summary Measures Value

Concordant 153240 48.9 Somers’ D 0.07

Discordant 130104 41.5 Goodman-Kruskal Gamma 0.08

Ties 30008 9.6 Kendall’s Tau-a 0.04

Total 313352 100.0

Association is between the response variable and predicted probabilities

Fits and Diagnostics for Unusual Observations

Observed

Obs Probability Fit SE Fit 95% CI Resid Std Resid Del Resid HI

1099 1.0000 0.6250 0.1210 (0.3772, 0.8210) 0.9695 1.00 0.99 0.0625

1100 1.0000 0.6250 0.1210 (0.3772, 0.8210) 0.9695 1.00 0.99 0.0625

1101 1.0000 0.6250 0.1210 (0.3772, 0.8210) 0.9695 1.00 0.99 0.0625

1102 1.0000 0.6250 0.1210 (0.3772, 0.8210) 0.9695 1.00 0.99 0.0625

1103 1.0000 0.6250 0.1210 (0.3772, 0.8210) 0.9695 1.00 0.99 0.0625

1104 1.0000 0.6250 0.1210 (0.3772, 0.8210) 0.9695 1.00 0.99 0.0625

1105 1.0000 0.6250 0.1210 (0.3772, 0.8210) 0.9695 1.00 0.99 0.0625

1106 1.0000 0.6250 0.1210 (0.3772, 0.8210) 0.9695 1.00 0.99 0.0625

1107 1.0000 0.6250 0.1210 (0.3772, 0.8210) 0.9695 1.00 0.99 0.0625

1108 1.0000 0.6250 0.1210 (0.3772, 0.8210) 0.9695 1.00 0.99 0.0625

1109 0.0000 0.6250 0.1210 (0.3772, 0.8210) -1.4006 -1.45 -1.44 0.0625

1110 0.0000 0.6250 0.1210 (0.3772, 0.8210) -1.4006 -1.45 -1.44 0.0625

1111 0.0000 0.6250 0.1210 (0.3772, 0.8210) -1.4006 -1.45 -1.44 0.0625

1112 0.0000 0.6250 0.1210 (0.3772, 0.8210) -1.4006 -1.45 -1.44 0.0625

1113 0.0000 0.6250 0.1210 (0.3772, 0.8210) -1.4006 -1.45 -1.44 0.0625

1114 0.0000 0.6250 0.1210 (0.3772, 0.8210) -1.4006 -1.45 -1.44 0.0625

1115 1.0000 0.7500 0.1531 (0.3771, 0.9370) 0.7585 0.81 0.79 0.1250

1116 1.0000 0.7500 0.1531 (0.3771, 0.9370) 0.7585 0.81 0.79 0.1250

1117 1.0000 0.7500 0.1531 (0.3771, 0.9370) 0.7585 0.81 0.79 0.1250

1118 1.0000 0.7500 0.1531 (0.3771, 0.9370) 0.7585 0.81 0.79 0.1250

1119 1.0000 0.7500 0.1531 (0.3771, 0.9370) 0.7585 0.81 0.79 0.1250

1120 1.0000 0.7500 0.1531 (0.3771, 0.9370) 0.7585 0.81 0.79 0.1250

1121 0.0000 0.7500 0.1531 (0.3771, 0.9370) -1.6651 -1.78 -1.79 0.1250

1122 0.0000 0.7500 0.1531 (0.3771, 0.9370) -1.6651 -1.78 -1.79 0.1250

Obs Cook’s D DFITS

1099 0.00 0.206559 X

1100 0.00 0.206559 X

1101 0.00 0.206559 X

1102 0.00 0.206559 X

1103 0.00 0.206559 X

1104 0.00 0.206559 X

1105 0.00 0.206559 X

1106 0.00 0.206559 X

1107 0.00 0.206559 X

1108 0.00 0.206559 X

1109 0.01 -0.344265 X

1110 0.01 -0.344265 X

1111 0.01 -0.344265 X

1112 0.01 -0.344265 X

1113 0.01 -0.344265 X

1114 0.01 -0.344265 X

1115 0.00 0.233285 X

1116 0.00 0.233285 X

1117 0.00 0.233285 X

1118 0.00 0.233285 X

1119 0.00 0.233285 X

1120 0.00 0.233285 X

1121 0.04 -0.699854 X

1122 0.04 -0.699854 X

X Unusual X

**10) Binary Logistic Regression: LEFT AND RIGHT LEG PREFERENT HORSES versus RELATIVE POSITION FROM INSIDE RAIL ON AC COURSES**

Method

Link function Logit

Categorical predictor coding (1, 0)

Rows used 1101

Response Information

Variable Value Count

LEFT/RIGHT LEAD A-C COURSES LEFT 544 (Event)

RIGHT 557

Total 1101

Deviance at Each Iterative Step

Step Deviance

1 1518.092404

2 1517.758782

3 1517.758749

4 1517.758749

Deviance Table

Source DF Seq Dev Contribution Adj Dev Adj Mean Chi- P-Value

Regression 12 8.40 0.55% 8.40 0.6998 8.40 0.753 FINNISHING POSITION A-C COURSES 12 8.40 0.55% 8.40 0.6998 8.40 0.753

Error 1088 1517.76 99.45% 1517.76 1.3950

Total 1100 1526.16 100.00%

Model Summary

Deviance Deviance

R-Sq R-Sq(adj) AIC

0.55% 0.00% 1543.76

Coefficients

Term Coef SE Coef 95% CI Z-Value P-Value VIF

Constant -0.103 0.227 (-0.547, 0.342) -0.45 0.651

FINNISHING POSITION A-C COURSES

POSITION ELEVEN 0.236 0.431 (-0.608, 1.080) 0.55 0.583 1.35

POSITION FIVE 0.185 0.291 (-0.385, 0.755) 0.64 0.524 2.27

POSITION FOUR 0.037 0.290 (-0.532, 0.606) 0.13 0.898 2.28

POSITION NINE 0.257 0.337 (-0.403, 0.917) 0.76 0.446 1.72

POSITION ONE 0.190 0.284 (-0.366, 0.746) 0.67 0.504 2.42

POSITION SEVEN 0.121 0.297 (-0.461, 0.703) 0.41 0.684 2.16

POSITION SIX 0.220 0.292 (-0.351, 0.792) 0.76 0.450 2.25

POSITION TEN -0.160 0.374 (-0.893, 0.573) -0.43 0.669 1.52

POSITION THIRTEEN 0.439 0.628 (-0.792, 1.670) 0.70 0.484 1.14

POSITION THREE -0.262 0.292 (-0.834, 0.310) -0.90 0.370 2.25

POSITION TWELVE -0.590 0.593 (-1.752, 0.571) -1.00 0.319 1.16

POSITION TWO 0.103 0.289 (-0.464, 0.670) 0.35 0.723 2.30

Odds Ratios for Categorical Predictors

Level A Level B Odds Ratio 95% CI

FINNISHING POSITION A-C COURSES

POSITION ELEVEN POSITION EIGHT 1.2664 (0.5446, 2.9446)

POSITION FIVE POSITION EIGHT 1.2036 (0.6808, 2.1280)

POSITION FOUR POSITION EIGHT 1.0378 (0.5875, 1.8329)

POSITION NINE POSITION EIGHT 1.2928 (0.6683, 2.5008)

POSITION ONE POSITION EIGHT 1.2088 (0.6933, 2.1077)

POSITION SEVEN POSITION EIGHT 1.1286 (0.6308, 2.0194)

POSITION SIX POSITION EIGHT 1.2466 (0.7036, 2.2086)

POSITION TEN POSITION EIGHT 0.8524 (0.4095, 1.7742)

POSITION THIRTEEN POSITION EIGHT 1.5514 (0.4531, 5.3112)

POSITION THREE POSITION EIGHT 0.7695 (0.4341, 1.3641)

POSITION TWELVE POSITION EIGHT 0.5541 (0.1734, 1.7707)

POSITION TWO POSITION EIGHT 1.1081 (0.6286, 1.9535)

POSITION FIVE POSITION ELEVEN 0.9504 (0.4266, 2.1175)

POSITION FOUR POSITION ELEVEN 0.8194 (0.3681, 1.8244)

POSITION NINE POSITION ELEVEN 1.0208 (0.4288, 2.4302)

POSITION ONE POSITION ELEVEN 0.9545 (0.4327, 2.1058)

POSITION SEVEN POSITION ELEVEN 0.8912 (0.3966, 2.0026)

POSITION SIX POSITION ELEVEN 0.9844 (0.4412, 2.1963)

POSITION TEN POSITION ELEVEN 0.6731 (0.2671, 1.6962)

POSITION THIRTEEN POSITION ELEVEN 1.2250 (0.3165, 4.7412)

POSITION THREE POSITION ELEVEN 0.6076 (0.2722, 1.3563)

POSITION TWELVE POSITION ELEVEN 0.4375 (0.1203, 1.5911)

POSITION TWO POSITION ELEVEN 0.8750 (0.3936, 1.9454)

POSITION FOUR POSITION FIVE 0.8622 (0.5212, 1.4262)

POSITION NINE POSITION FIVE 1.0741 (0.5870, 1.9653)

POSITION ONE POSITION FIVE 1.0043 (0.6161, 1.6371)

POSITION SEVEN POSITION FIVE 0.9377 (0.5587, 1.5739)

POSITION SIX POSITION FIVE 1.0357 (0.6240, 1.7191)

POSITION TEN POSITION FIVE 0.7082 (0.3576, 1.4026)

POSITION THIRTEEN POSITION FIVE 1.2889 (0.3875, 4.2869)

POSITION THREE POSITION FIVE 0.6393 (0.3849, 1.0618)

POSITION TWELVE POSITION FIVE 0.4603 (0.1485, 1.4267)

POSITION TWO POSITION FIVE 0.9206 (0.5578, 1.5196)

POSITION NINE POSITION FOUR 1.2458 (0.6815, 2.2773)

POSITION ONE POSITION FOUR 1.1649 (0.7154, 1.8967)

POSITION SEVEN POSITION FOUR 1.0876 (0.6487, 1.8235)

POSITION SIX POSITION FOUR 1.2013 (0.7245, 1.9917)

POSITION TEN POSITION FOUR 0.8214 (0.4151, 1.6255)

POSITION THIRTEEN POSITION FOUR 1.4949 (0.4497, 4.9698)

POSITION THREE POSITION FOUR 0.7415 (0.4470, 1.2302)

POSITION TWELVE POSITION FOUR 0.5339 (0.1723, 1.6540)

POSITION TWO POSITION FOUR 1.0678 (0.6476, 1.7605)

POSITION ONE POSITION NINE 0.9351 (0.5178, 1.6886)

POSITION SEVEN POSITION NINE 0.8730 (0.4718, 1.6155)

POSITION SIX POSITION NINE 0.9643 (0.5260, 1.7678)

POSITION TEN POSITION NINE 0.6593 (0.3083, 1.4099)

POSITION THIRTEEN POSITION NINE 1.2000 (0.3449, 4.1756)

POSITION THREE POSITION NINE 0.5952 (0.3245, 1.0918)

POSITION TWELVE POSITION NINE 0.4286 (0.1318, 1.3935)

POSITION TWO POSITION NINE 0.8571 (0.4697, 1.5641)

POSITION SEVEN POSITION ONE 0.9336 (0.5648, 1.5432)

POSITION SIX POSITION ONE 1.0312 (0.6311, 1.6851)

POSITION TEN POSITION ONE 0.7051 (0.3602, 1.3805)

POSITION THIRTEEN POSITION ONE 1.2833 (0.3884, 4.2406)

POSITION THREE POSITION ONE 0.6366 (0.3893, 1.0408)

POSITION TWELVE POSITION ONE 0.4583 (0.1489, 1.4107)

POSITION TWO POSITION ONE 0.9167 (0.5642, 1.4892)

POSITION SIX POSITION SEVEN 1.1045 (0.6566, 1.8582)

POSITION TEN POSITION SEVEN 0.7552 (0.3775, 1.5109)

POSITION THIRTEEN POSITION SEVEN 1.3745 (0.4109, 4.5980)

POSITION THREE POSITION SEVEN 0.6818 (0.4051, 1.1477)

POSITION TWELVE POSITION SEVEN 0.4909 (0.1574, 1.5308)

POSITION TWO POSITION SEVEN 0.9818 (0.5868, 1.6427)

POSITION TEN POSITION SIX 0.6838 (0.3446, 1.3566)

POSITION THIRTEEN POSITION SIX 1.2444 (0.3738, 4.1431)

POSITION THREE POSITION SIX 0.6173 (0.3708, 1.0276)

POSITION TWELVE POSITION SIX 0.4444 (0.1432, 1.3790)

POSITION TWO POSITION SIX 0.8889 (0.5373, 1.4706)

POSITION THIRTEEN POSITION TEN 1.8200 (0.5024, 6.5932)

POSITION THREE POSITION TEN 0.9028 (0.4548, 1.7919)

POSITION TWELVE POSITION TEN 0.6500 (0.1916, 2.2052)

POSITION TWO POSITION TEN 1.3000 (0.6580, 2.5686)

POSITION THREE POSITION THIRTEEN 0.4960 (0.1490, 1.6519)

POSITION TWELVE POSITION THIRTEEN 0.3571 (0.0742, 1.7192)

POSITION TWO POSITION THIRTEEN 0.7143 (0.2151, 2.3725)

POSITION TWELVE POSITION THREE 0.7200 (0.2320, 2.2345)

POSITION TWO POSITION THREE 1.4400 (0.8698, 2.3839)

POSITION TWO POSITION TWELVE 2.0000 (0.6462, 6.1898)

Odds ratio for level A relative to level B

Regression Equation

P(LEFT) = exp(Y')/(1 + exp(Y'))

Y' = -0.103 + 0.0 FINNISHING POSITION A-C COURSES_POSITION EIGHT

+ 0.236 FINNISHING POSITION A-C COURSES_POSITION ELEVEN

+ 0.185 FINNISHING POSITION A-C COURSES_POSITION FIVE

+ 0.037 FINNISHING POSITION A-C COURSES_POSITION FOUR

+ 0.257 FINNISHING POSITION A-C COURSES_POSITION NINE

+ 0.190 FINNISHING POSITION A-C COURSES_POSITION ONE

+ 0.121 FINNISHING POSITION A-C COURSES_POSITION SEVEN

+ 0.220 FINNISHING POSITION A-C COURSES_POSITION SIX

- 0.160 FINNISHING POSITION A-C COURSES_POSITION TEN

+ 0.439 FINNISHING POSITION A-C COURSES_POSITION THIRTEEN

- 0.262 FINNISHING POSITION A-C COURSES_POSITION THREE

- 0.590 FINNISHING POSITION A-C COURSES_POSITION TWELVE

+ 0.103 FINNISHING POSITION A-C COURSES_POSITION TWO

Goodness-of-Fit Tests

Test DF Chi-Square P-Value

Deviance 1088 1517.76 0.000

Pearson 1088 1101.00 0.385

Hosmer-Lemeshow 6 0.00 1.000

Measures of Association

Pairs Number Percent Summary Measures Value

Concordant 146159 48.2 Somers’ D 0.09

Discordant 119054 39.3 Goodman-Kruskal Gamma 0.10

Ties 37795 12.5 Kendall’s Tau-a 0.04

Total 303008 100.0

Association is between the response variable and predicted probabilities

Fits and Diagnostics for Unusual Observations

Observed

Obs Probability Fit SE Fit 95% CI Resid Std Resid Del Resid HI

1075 1.0000 0.3333 0.1217 (0.1460, 0.5940) 1.4823 1.53 1.53 0.0666667

1076 1.0000 0.3333 0.1217 (0.1460, 0.5940) 1.4823 1.53 1.53 0.0666667

1077 1.0000 0.3333 0.1217 (0.1460, 0.5940) 1.4823 1.53 1.53 0.0666667

1078 1.0000 0.3333 0.1217 (0.1460, 0.5940) 1.4823 1.53 1.53 0.0666667

1079 1.0000 0.3333 0.1217 (0.1460, 0.5940) 1.4823 1.53 1.53 0.0666667

1080 0.0000 0.3333 0.1217 (0.1460, 0.5940) -0.9005 -0.93 -0.92 0.0666667

1081 0.0000 0.3333 0.1217 (0.1460, 0.5940) -0.9005 -0.93 -0.92 0.0666667

1082 0.0000 0.3333 0.1217 (0.1460, 0.5940) -0.9005 -0.93 -0.92 0.0666667

1083 0.0000 0.3333 0.1217 (0.1460, 0.5940) -0.9005 -0.93 -0.92 0.0666667

1084 0.0000 0.3333 0.1217 (0.1460, 0.5940) -0.9005 -0.93 -0.92 0.0666667

1085 0.0000 0.3333 0.1217 (0.1460, 0.5940) -0.9005 -0.93 -0.92 0.0666667

1086 0.0000 0.3333 0.1217 (0.1460, 0.5940) -0.9005 -0.93 -0.92 0.0666667

1087 0.0000 0.3333 0.1217 (0.1460, 0.5940) -0.9005 -0.93 -0.92 0.0666667

1088 0.0000 0.3333 0.1217 (0.1460, 0.5940) -0.9005 -0.93 -0.92 0.0666667

1089 0.0000 0.3333 0.1217 (0.1460, 0.5940) -0.9005 -0.93 -0.92 0.0666667

1090 1.0000 0.5833 0.1423 (0.3076, 0.8152) 1.0383 1.08 1.07 0.0833333

1091 1.0000 0.5833 0.1423 (0.3076, 0.8152) 1.0383 1.08 1.07 0.0833333

1092 1.0000 0.5833 0.1423 (0.3076, 0.8152) 1.0383 1.08 1.07 0.0833333

1093 1.0000 0.5833 0.1423 (0.3076, 0.8152) 1.0383 1.08 1.07 0.0833333

1094 1.0000 0.5833 0.1423 (0.3076, 0.8152) 1.0383 1.08 1.07 0.0833333

1095 1.0000 0.5833 0.1423 (0.3076, 0.8152) 1.0383 1.08 1.07 0.0833333

1096 1.0000 0.5833 0.1423 (0.3076, 0.8152) 1.0383 1.08 1.07 0.0833333

1097 0.0000 0.5833 0.1423 (0.3076, 0.8152) -1.3232 -1.38 -1.37 0.0833333

1098 0.0000 0.5833 0.1423 (0.3076, 0.8152) -1.3232 -1.38 -1.37 0.0833333

1099 0.0000 0.5833 0.1423 (0.3076, 0.8152) -1.3232 -1.38 -1.37 0.0833333

1100 0.0000 0.5833 0.1423 (0.3076, 0.8152) -1.3232 -1.38 -1.37 0.0833333

1101 0.0000 0.5833 0.1423 (0.3076, 0.8152) -1.3232 -1.38 -1.37 0.0833333

Obs Cook’s D DFITS

1075 0.01 0.391230 X

1076 0.01 0.391230 X

1077 0.01 0.391230 X

1078 0.01 0.391230 X

1079 0.01 0.391230 X

1080 0.00 -0.195615 X

1081 0.00 -0.195615 X

1082 0.00 -0.195615 X

1083 0.00 -0.195615 X

1084 0.00 -0.195615 X

1085 0.00 -0.195615 X

1086 0.00 -0.195615 X

1087 0.00 -0.195615 X

1088 0.00 -0.195615 X

1089 0.00 -0.195615 X

1090 0.01 0.266155 X

1091 0.01 0.266155 X

1092 0.01 0.266155 X

1093 0.01 0.266155 X

1094 0.01 0.266155 X

1095 0.01 0.266155 X

1096 0.01 0.266155 X

1097 0.01 -0.372616 X

1098 0.01 -0.372616 X

1099 0.01 -0.372616 X

1100 0.01 -0.372616 X

1101 0.01 -0.372616 X

X Unusual X

**11) Binary Logistic Regression: LEFT AND RIGHT LEG PREFERENT HORSES versus AGE (YEARS)**

Method

Link function Logit

Categorical predictor coding (1, 0)

Rows used 2092

Response Information

Variable Value Count

LEFT/RIGHT LEG LEAD GENDER FREE LEFT 1072 (Event)

RIGHT 1020

Total 2092

Deviance at Each Iterative Step

Step Deviance

1 2894.397310

2 2894.142793

3 2894.142782

4 2894.142782

Deviance Table

Source DF Seq Dev Contribution Adj Dev Adj Mean Chi-Square P-Value

Regression 9 4.69 0.16% 4.69 0.5214 4.69 0.860

AGE (YEARS) 9 4.69 0.16% 4.69 0.5214 4.69 0.860

Error 2082 2894.14 99.84% 2894.14 1.3901

Total 2091 2898.84 100.00%

Model Summary

Deviance Deviance

R-Sq R-Sq(adj) AIC

0.16% 0.00% 2914.14

Coefficients

Term Coef SE Coef 95% CI Z-Value P-Value VIF

Constant 0.0781 0.0825 (-0.0835, 0.2398) 0.95 0.343

AGE (YEARS)

3 -0.086 0.119 ( -0.319, 0.148) -0.72 0.473 1.42

4 0.024 0.132 ( -0.236, 0.284) 0.18 0.857 1.34

5 -0.048 0.165 ( -0.370, 0.275) -0.29 0.772 1.21

6 -0.180 0.190 ( -0.553, 0.192) -0.95 0.342 1.15

7 0.162 0.210 ( -0.250, 0.573) 0.77 0.441 1.12

8 0.128 0.242 ( -0.347, 0.602) 0.53 0.598 1.09

9 -0.078 0.319 ( -0.704, 0.548) -0.24 0.807 1.05

10 -0.617 0.483 ( -1.563, 0.329) -1.28 0.201 1.02

11 -0.078 0.821 ( -1.687, 1.530) -0.10 0.924 1.01

Odds Ratios for Categorical Predictors

Level A Level B Odds Ratio 95% CI

AGE (YEARS)

3 2 0.9180 (0.7269, 1.1594)

4 2 1.0241 (0.7899, 1.3278)

5 2 0.9534 (0.6905, 1.3166)

6 2 0.8349 (0.5754, 1.2115)

7 2 1.1753 (0.7790, 1.7732)

8 2 1.1362 (0.7069, 1.8263)

9 2 0.9248 (0.4945, 1.7297)

10 2 0.5395 (0.2095, 1.3895)

11 2 0.9248 (0.1852, 4.6196)

4 3 1.1155 (0.8568, 1.4525)

5 3 1.0386 (0.7495, 1.4390)

6 3 0.9095 (0.6249, 1.3236)

7 3 1.2802 (0.8463, 1.9367)

8 3 1.2377 (0.7682, 1.9940)

9 3 1.0074 (0.5377, 1.8875)

10 3 0.5877 (0.2279, 1.5153)

11 3 1.0074 (0.2015, 5.0355)

5 4 0.9310 (0.6591, 1.3151)

6 4 0.8153 (0.5508, 1.2067)

7 4 1.1476 (0.7471, 1.7630)

8 4 1.1095 (0.6795, 1.8116)

9 4 0.9031 (0.4771, 1.7094)

10 4 0.5268 (0.2029, 1.3676)

11 4 0.9031 (0.1799, 4.5321)

6 5 0.8757 (0.5660, 1.3549)

7 5 1.2327 (0.7703, 1.9726)

8 5 1.1917 (0.7040, 2.0174)

9 5 0.9700 (0.4982, 1.8885)

10 5 0.5658 (0.2138, 1.4973)

11 5 0.9700 (0.1911, 4.9236)

7 6 1.4077 (0.8492, 2.3335)

8 6 1.3609 (0.7788, 2.3781)

9 6 1.1077 (0.5547, 2.2120)

10 6 0.6462 (0.2399, 1.7401)

11 6 1.1077 (0.2159, 5.6822)

8 7 0.9667 (0.5386, 1.7351)

9 7 0.7869 (0.3856, 1.6059)

10 7 0.4590 (0.1679, 1.2552)

11 7 0.7869 (0.1520, 4.0743)

9 8 0.8140 (0.3839, 1.7259)

10 8 0.4748 (0.1689, 1.3345)

11 8 0.8140 (0.1546, 4.2866)

10 9 0.5833 (0.1920, 1.7722)

11 9 1.0000 (0.1807, 5.5334)

11 10 1.7143 (0.2690, 10.9243)

Odds ratio for level A relative to level B

Regression Equation

P(LEFT) = exp(Y')/(1 + exp(Y'))

Y' = 0.0781 + 0.0 AGE (YEARS)_2 - 0.086 AGE (YEARS)_3 + 0.024 AGE (YEARS)_4

- 0.048 AGE (YEARS)_5 - 0.180 AGE (YEARS)_6 + 0.162 AGE (YEARS)_7 + 0.128 AGE (YEARS)_8

- 0.078 AGE (YEARS)_9 - 0.617 AGE (YEARS)_10 - 0.078 AGE (YEARS)_11

Goodness-of-Fit Tests

Test DF Chi-Square P-Value

Deviance 2082 2894.14 0.000

Pearson 2082 2092.00 0.434

Hosmer-Lemeshow 3 0.00 1.000

Measures of Association

Pairs Number Percent Summary Measures Value

Concordant 464509 42.5 Somers’ D 0.05

Discordant 414728 37.9 Goodman-Kruskal Gamma 0.06

Ties 214203 19.6 Kendall’s Tau-a 0.02

Total 1093440 100.0

Association is between the response variable and predicted probabilities

Fits and Diagnostics for Unusual Observations

Observed

Obs Probability Fit SE Fit 95% CI Resid Std Resid Del Resid HI

2026 1.000 0.500 0.077 (0.353, 0.647) 1.177 1.19 1.19 0.023810

2027 1.000 0.500 0.077 (0.353, 0.647) 1.177 1.19 1.19 0.023810

2028 1.000 0.500 0.077 (0.353, 0.647) 1.177 1.19 1.19 0.023810

2029 1.000 0.500 0.077 (0.353, 0.647) 1.177 1.19 1.19 0.023810

2030 1.000 0.500 0.077 (0.353, 0.647) 1.177 1.19 1.19 0.023810

2031 1.000 0.500 0.077 (0.353, 0.647) 1.177 1.19 1.19 0.023810

2032 1.000 0.500 0.077 (0.353, 0.647) 1.177 1.19 1.19 0.023810

2033 1.000 0.500 0.077 (0.353, 0.647) 1.177 1.19 1.19 0.023810

2034 1.000 0.500 0.077 (0.353, 0.647) 1.177 1.19 1.19 0.023810

2035 1.000 0.500 0.077 (0.353, 0.647) 1.177 1.19 1.19 0.023810

2036 1.000 0.500 0.077 (0.353, 0.647) 1.177 1.19 1.19 0.023810

2037 1.000 0.500 0.077 (0.353, 0.647) 1.177 1.19 1.19 0.023810

2038 1.000 0.500 0.077 (0.353, 0.647) 1.177 1.19 1.19 0.023810

2039 1.000 0.500 0.077 (0.353, 0.647) 1.177 1.19 1.19 0.023810

2040 1.000 0.500 0.077 (0.353, 0.647) 1.177 1.19 1.19 0.023810

2041 1.000 0.500 0.077 (0.353, 0.647) 1.177 1.19 1.19 0.023810

2042 1.000 0.500 0.077 (0.353, 0.647) 1.177 1.19 1.19 0.023810

2043 1.000 0.500 0.077 (0.353, 0.647) 1.177 1.19 1.19 0.023810

2044 1.000 0.500 0.077 (0.353, 0.647) 1.177 1.19 1.19 0.023810

2045 1.000 0.500 0.077 (0.353, 0.647) 1.177 1.19 1.19 0.023810

2046 1.000 0.500 0.077 (0.353, 0.647) 1.177 1.19 1.19 0.023810

2047 0.000 0.500 0.077 (0.353, 0.647) -1.177 -1.19 -1.19 0.023810

2048 0.000 0.500 0.077 (0.353, 0.647) -1.177 -1.19 -1.19 0.023810

2049 0.000 0.500 0.077 (0.353, 0.647) -1.177 -1.19 -1.19 0.023810

2050 0.000 0.500 0.077 (0.353, 0.647) -1.177 -1.19 -1.19 0.023810

2051 0.000 0.500 0.077 (0.353, 0.647) -1.177 -1.19 -1.19 0.023810

2052 0.000 0.500 0.077 (0.353, 0.647) -1.177 -1.19 -1.19 0.023810

2053 0.000 0.500 0.077 (0.353, 0.647) -1.177 -1.19 -1.19 0.023810

2054 0.000 0.500 0.077 (0.353, 0.647) -1.177 -1.19 -1.19 0.023810

2055 0.000 0.500 0.077 (0.353, 0.647) -1.177 -1.19 -1.19 0.023810

2056 0.000 0.500 0.077 (0.353, 0.647) -1.177 -1.19 -1.19 0.023810

2057 0.000 0.500 0.077 (0.353, 0.647) -1.177 -1.19 -1.19 0.023810

2058 0.000 0.500 0.077 (0.353, 0.647) -1.177 -1.19 -1.19 0.023810

2059 0.000 0.500 0.077 (0.353, 0.647) -1.177 -1.19 -1.19 0.023810

2060 0.000 0.500 0.077 (0.353, 0.647) -1.177 -1.19 -1.19 0.023810

2061 0.000 0.500 0.077 (0.353, 0.647) -1.177 -1.19 -1.19 0.023810

2062 0.000 0.500 0.077 (0.353, 0.647) -1.177 -1.19 -1.19 0.023810

2063 0.000 0.500 0.077 (0.353, 0.647) -1.177 -1.19 -1.19 0.023810

2064 0.000 0.500 0.077 (0.353, 0.647) -1.177 -1.19 -1.19 0.023810

2065 0.000 0.500 0.077 (0.353, 0.647) -1.177 -1.19 -1.19 0.023810

2066 0.000 0.500 0.077 (0.353, 0.647) -1.177 -1.19 -1.19 0.023810

2067 0.000 0.500 0.077 (0.353, 0.647) -1.177 -1.19 -1.19 0.023810

2068 1.000 0.368 0.111 (0.187, 0.597) 1.413 1.45 1.45 0.052632

2069 1.000 0.368 0.111 (0.187, 0.597) 1.413 1.45 1.45 0.052632

2070 1.000 0.368 0.111 (0.187, 0.597) 1.413 1.45 1.45 0.052632

2071 1.000 0.368 0.111 (0.187, 0.597) 1.413 1.45 1.45 0.052632

2072 1.000 0.368 0.111 (0.187, 0.597) 1.413 1.45 1.45 0.052632

2073 1.000 0.368 0.111 (0.187, 0.597) 1.413 1.45 1.45 0.052632

2074 1.000 0.368 0.111 (0.187, 0.597) 1.413 1.45 1.45 0.052632

2075 0.000 0.368 0.111 (0.187, 0.597) -0.959 -0.98 -0.98 0.052632

2076 0.000 0.368 0.111 (0.187, 0.597) -0.959 -0.98 -0.98 0.052632

2077 0.000 0.368 0.111 (0.187, 0.597) -0.959 -0.98 -0.98 0.052632

2078 0.000 0.368 0.111 (0.187, 0.597) -0.959 -0.98 -0.98 0.052632

2079 0.000 0.368 0.111 (0.187, 0.597) -0.959 -0.98 -0.98 0.052632

2080 0.000 0.368 0.111 (0.187, 0.597) -0.959 -0.98 -0.98 0.052632

2081 0.000 0.368 0.111 (0.187, 0.597) -0.959 -0.98 -0.98 0.052632

2082 0.000 0.368 0.111 (0.187, 0.597) -0.959 -0.98 -0.98 0.052632

2083 0.000 0.368 0.111 (0.187, 0.597) -0.959 -0.98 -0.98 0.052632

2084 0.000 0.368 0.111 (0.187, 0.597) -0.959 -0.98 -0.98 0.052632

2085 0.000 0.368 0.111 (0.187, 0.597) -0.959 -0.98 -0.98 0.052632

2086 0.000 0.368 0.111 (0.187, 0.597) -0.959 -0.98 -0.98 0.052632

2087 1.000 0.500 0.204 (0.168, 0.832) 1.177 1.29 1.26 0.166667

2088 1.000 0.500 0.204 (0.168, 0.832) 1.177 1.29 1.26 0.166667

2089 1.000 0.500 0.204 (0.168, 0.832) 1.177 1.29 1.26 0.166667

2090 0.000 0.500 0.204 (0.168, 0.832) -1.177 -1.29 -1.26 0.166667

2091 0.000 0.500 0.204 (0.168, 0.832) -1.177 -1.29 -1.26 0.166667

2092 0.000 0.500 0.204 (0.168, 0.832) -1.177 -1.29 -1.26 0.166667

Obs Cook’s D DFITS

2026 0.00 0.158067 X

2027 0.00 0.158067 X

2028 0.00 0.158067 X

2029 0.00 0.158067 X

2030 0.00 0.158067 X

2031 0.00 0.158067 X

2032 0.00 0.158067 X

2033 0.00 0.158067 X

2034 0.00 0.158067 X

2035 0.00 0.158067 X

2036 0.00 0.158067 X

2037 0.00 0.158067 X

2038 0.00 0.158067 X

2039 0.00 0.158067 X

2040 0.00 0.158067 X

2041 0.00 0.158067 X

2042 0.00 0.158067 X

2043 0.00 0.158067 X

2044 0.00 0.158067 X

2045 0.00 0.158067 X

2046 0.00 0.158067 X

2047 0.00 -0.158067 X

2048 0.00 -0.158067 X

2049 0.00 -0.158067 X

2050 0.00 -0.158067 X

2051 0.00 -0.158067 X

2052 0.00 -0.158067 X

2053 0.00 -0.158067 X

2054 0.00 -0.158067 X

2055 0.00 -0.158067 X

2056 0.00 -0.158067 X

2057 0.00 -0.158067 X

2058 0.00 -0.158067 X

2059 0.00 -0.158067 X

2060 0.00 -0.158067 X

2061 0.00 -0.158067 X

2062 0.00 -0.158067 X

2063 0.00 -0.158067 X

2064 0.00 -0.158067 X

2065 0.00 -0.158067 X

2066 0.00 -0.158067 X

2067 0.00 -0.158067 X

2068 0.01 0.317063 X

2069 0.01 0.317063 X

2070 0.01 0.317063 X

2071 0.01 0.317063 X

2072 0.01 0.317063 X

2073 0.01 0.317063 X

2074 0.01 0.317063 X

2075 0.00 -0.184954 X

2076 0.00 -0.184954 X

2077 0.00 -0.184954 X

2078 0.00 -0.184954 X

2079 0.00 -0.184954 X

2080 0.00 -0.184954 X

2081 0.00 -0.184954 X

2082 0.00 -0.184954 X

2083 0.00 -0.184954 X

2084 0.00 -0.184954 X

2085 0.00 -0.184954 X

2086 0.00 -0.184954 X

2087 0.02 0.489898 X

2088 0.02 0.489898 X

2089 0.02 0.489898 X

2090 0.02 -0.489898 X

2091 0.02 -0.489898 X

2092 0.02 -0.489898 X

X Unusual X

**12) Test and CI for One Proportion: RIGHT VS LEFT LEG PREFERENT HORSES, STALLIONS**

Test of p = 0.5 vs p ≠ 0.5

Event = RIGHT

Variable X N Sample p 90% CI Z-Value P-Value

LEFT/RIGHT STALLIONS 135 263 0.513308 (0.462613, 0.564003) 0.43 0.666

Using the normal approximation.

**13) Test and CI for One Proportion: RIGHT VS LEFT LEG PREFERENT HORSES, GELDINGS**

Test of p = 0.5 vs p ≠ 0.5

Event = RIGHT

Variable X N Sample p 90% CI Z-Value P-Value

LEFT/RIGHT GELDINGS 578 1177 0.491079 (0.467111, 0.515047) -0.61 0.540

Using the normal approximation.

**14) Test and CI for One Proportion: RIGHT VS LEFT LEG PREFERENT HORSES, MARES**

Test of p = 0.5 vs p ≠ 0.5

Event = RIGHT

Variable X N Sample p 90% CI Z-Value P-Value

LEFT/RIGHT MARES 308 655 0.470229 (0.438151, 0.502307) -1.52 0.128

Using the normal approximation.

**15) Test and CI for One Proportion: RIGHT VS LEFT LEG PREFERENT HORSES. MALES**

Test of p = 0.5 vs p ≠ 0.5

Event = RIGHT

Variable X N Sample p 90% CI Z-Value P-Value

LEFT/RIGHT MALE 713 1440 0.495139 (0.473467, 0.516811) -0.37 0.712

Using the normal approximation.

**16) Binary Logistic Regression: LEFT VS RIGHT LEG PREFERENT HORSES versus SEX (MALE OR FEMALE)**

Method

Link function Logit

Categorical predictor coding (1, 0)

Rows used 2095

Response Information

Variable Value Count

LEFT/RIGHT MALE FEMALE LEFT 1074 (Event)

RIGHT 1021

Total 2095

Deviance at Each Iterative Step

Step Deviance

1 2901.940101

2 2901.827062

3 2901.827062

Deviance Table

Source DF Seq Dev Contribution Adj Dev Adj Mean Chi-Square P-Value

Regression 1 1.12 0.04% 1.12 1.119 1.12 0.290

SEX MALE FEMALE 1 1.12 0.04% 1.12 1.119 1.12 0.290

Error 2093 2901.83 99.96% 2901.83 1.386

Total 2094 2902.95 100.00%

Model Summary

Deviance Deviance

R-Sq R-Sq(adj) AIC

0.04% 0.00% 2905.83

Coefficients

Term Coef SE Coef 95% CI Z-Value P-Value VIF

Constant 0.1192 0.0783 (-0.0342, 0.2727) 1.52 0.128

SEX MALE FEMALE

MALE -0.0998 0.0944 (-0.2848, 0.0852) -1.06 0.290 1.00

Odds Ratios for Categorical Predictors

Level A Level B Odds Ratio 95% CI

SEX MALE FEMALE

MALE FEMALE 0.9050 (0.7522, 1.0889)

Odds ratio for level A relative to level B

Regression Equation

P(LEFT) = exp(Y')/(1 + exp(Y'))

Y' = 0.1192 + 0.0 SEX MALE FEMALE_FEMALE - 0.0998 SEX MALE FEMALE_MALE

Goodness-of-Fit Tests

Test DF Chi-Square P-Value

Deviance 2093 2901.83 0.000

Pearson 2093 2095.00 0.484

Hosmer-Lemeshow 0 0.00 *

Measures of Association

Pairs Number Percent Summary Measures Value

Concordant 247411 22.6 Somers’ D 0.02

Discordant 223916 20.4 Goodman-Kruskal Gamma 0.05

Ties 625227 57.0 Kendall’s Tau-a 0.01

Total 1096554 100.0

Association is between the response variable and predicted probabilities

**17) Binary Logistic Regression: LEFT AND RIGHT LEG PREFERENT HORSES versus SEX (STALLIONS = S, GELDINGS=G AND MARES =M)**

Method

Link function Logit

Categorical predictor coding (1, 0)

Rows used 2095

Response Information

Variable Value Count

LEFT/RIGHT S G M LEFT 1074 (Event)

RIGHT 1021

Total 2095

Deviance at Each Iterative Step

Step Deviance

1 2901.534950

2 2901.402140

3 2901.402140

Deviance Table

Source DF Seq Dev Contribution Adj Dev Adj Mean Chi-Square

Regression 2 1.54 0.05% 1.54 0.7718 1.54

SEX / STALLION = S GELD=G MARE 2 1.54 0.05% 1.54 0.7718 1.54

Error 2092 2901.40 99.95% 2901.40 1.3869

Total 2094 2902.95 100.00%

Source P-Value

Regression 0.462

SEX / STALLION = S GELD=G MARE 0.462

Error

Total

Model Summary

Deviance Deviance

R-Sq R-Sq(adj) AIC

0.05% 0.00% 2907.40

Coefficients

Term Coef SE Coef 95% CI Z-Value P-Value VIF

Constant 0.0357 0.0583 (-0.0786, 0.1500) 0.61 0.540

SEX / STALLION = S GELD=G MARE

M 0.0835 0.0976 (-0.1078, 0.2749) 0.86 0.392 1.07

S -0.089 0.136 ( -0.356, 0.179) -0.65 0.515 1.07

Odds Ratios for Categorical Predictors

Level A Level B Odds Ratio 95% CI

SEX / STALLION = S GELD=G MARE

M G 1.0871 (0.8978, 1.3163)

S G 0.9149 (0.7002, 1.1954)

S M 0.8416 (0.6320, 1.1206)

Odds ratio for level A relative to level B

Regression Equation

P(LEFT) = exp(Y')/(1 + exp(Y'))

Y' = 0.0357 + 0.0 SEX / STALLION = S GELD=G MARE_G + 0.0835 SEX / STALLION = S GELD=G MARE_M

- 0.089 SEX / STALLION = S GELD=G MARE_S

Goodness-of-Fit Tests

Test DF Chi-Square P-Value

Deviance 2092 2901.40 0.000

Pearson 2092 2095.00 0.477

Hosmer-Lemeshow 1 0.00 1.000

Measures of Association

Pairs Number Percent Summary Measures Value

Concordant 328276 29.9 Somers’ D 0.03

Discordant 297900 27.2 Goodman-Kruskal Gamma 0.05

Ties 470378 42.9 Kendall’s Tau-a 0.01

Total 1096554 100.0

Association is between the response variable and predicted probability

**18) HISTOGRAMS AND EQUAL VARIANCE TEST RESULTS GENERATED FROM LI DATA CONTAINED IN SEPARATE EXCEL SPREAD SHEET**

A) ALL HORSES

B) FEMALE HORSES

C) MALE HORSES

D) GELDINGS

E) STALLIONS

**19) FURTHER TEST OF POPULATION LATERALITY USING LI DATA.**

A) NORMALITY TEST AND JOHNSON TRANSFORMATION OF LI DATA

1. One-Sample T: NORMALISED TRANSFORMED LI DATA

Test of μ = 0 vs ≠ 0

Variable N Mean StDev SE Mean 95% CI T P

NORMALISED LI DATA_1 44 0.048 0.998 0.150 (-0.255, 0.351) 0.32 0.751

**20) ONE PROPORTION Z TESTS FOR SIGNIFICANCE OF LATERALITY OF INDIVIDUAL HORSES USING REPEATED MEASURES DATA**

Test and CI for One Proportion: (A)

Test of p = 0.5 vs p ≠ 0.5

Event = 2

Variable X N Sample p 95% CI Z-Value P-Value

(A) 20 28 0.714286 (0.546957, 0.881615) 2.27 0.023

Using the normal approximation.

Test and CI for One Proportion: (B)

Test of p = 0.5 vs p ≠ 0.5

Event = 2

Exact

Variable X N Sample p 95% CI P-Value

(B) 14 18 0.777778 (0.523627, 0.935908) 0.031

Test and CI for One Proportion: (C)

Test of p = 0.5 vs p ≠ 0.5

Event = 2

Exact

Variable X N Sample p 95% CI P-Value

(C) 4 21 0.190476 (0.054464, 0.419066) 0.007

Test and CI for One Proportion: (D)

Test of p = 0.5 vs p ≠ 0.5

Event = 2

Variable X N Sample p 95% CI Z-Value P-Value

(D) 8 14 0.571429 (0.312204, 0.830653) 0.53 0.593

Using the normal approximation.

Test and CI for One Proportion: (E)

Test of p = 0.5 vs p ≠ 0.5

Event = 2

Exact

Variable X N Sample p 95% CI P-Value

(E) 6 8 0.750000 (0.349144, 0.968146) 0.289

Test and CI for One Proportion: (F)

Test of p = 0.5 vs p ≠ 0.5

Event = 1

Exact

Variable X N Sample p 95% Lower Bound P-Value

(F) 7 7 1.000000 0.651836 0.016

Test and CI for One Proportion: (G)

Test of p = 0.5 vs p ≠ 0.5

Event = 2

Exact

Variable X N Sample p 95% CI P-Value

(G) 1 7 0.142857 (0.003610, 0.578723) 0.125

Test and CI for One Proportion: (H)

Test of p = 0.5 vs p ≠ 0.5

Event = 2

Exact

Variable X N Sample p 95% CI P-Value

(H) 7 9 0.777778 (0.399906, 0.971855) 0.180

Test and CI for One Proportion: (I)

Test of p = 0.5 vs p ≠ 0.5

Event = 2

Variable X N Sample p 95% CI Z-Value P-Value

(I) 5 17 0.294118 (0.077522, 0.510714) -1.70 0.090

Using the normal approximation.

Test and CI for One Proportion: (J)

Test of p = 0.5 vs p ≠ 0.5

Event = 2

Exact

Variable X N Sample p 95% CI P-Value

(J) 3 16 0.187500 (0.040474, 0.456457) 0.021

Test and CI for One Proportion: (K)

Test of p = 0.5 vs p ≠ 0.5

Event = 2

Variable X N Sample p 95% CI Z-Value P-Value

(K) 3 8 0.375000 (0.039526, 0.710474) -0.71 0.480

Using the normal approximation.

Test and CI for One Proportion: (L)

Test of p = 0.5 vs p ≠ 0.5

Event = 2

Variable X N Sample p 95% CI Z-Value P-Value

(L) 10 31 0.322581 (0.158024, 0.487137) -1.98 0.048

Using the normal approximation.

Test and CI for One Proportion: (M)

Test of p = 0.5 vs p ≠ 0.5

Event = 2

Variable X N Sample p 95% CI Z-Value P-Value

(M) 7 12 0.583333 (0.304394, 0.862273) 0.58 0.564

Using the normal approximation.

Test and CI for One Proportion: (N)

Test of p = 0.5 vs p ≠ 0.5

Event = 2

Variable X N Sample p 95% CI Z-Value P-Value

(N) 10 20 0.500000 (0.280869, 0.719131) 0.00 1.000

Using the normal approximation.

Test and CI for One Proportion: (O)

Test of p = 0.5 vs p ≠ 0.5

Event = 2

Exact

Variable X N Sample p 95% CI P-Value

(O) 2 12 0.166667 (0.020863, 0.484138) 0.039

Test and CI for One Proportion: (P)

Test of p = 0.5 vs p ≠ 0.5

Event = 2

Exact

Variable X N Sample p 95% CI P-Value

(P) 10 14 0.714286 (0.418965, 0.916111) 0.180

Test and CI for One Proportion: (Q)

Test of p = 0.5 vs p ≠ 0.5

Event = 2

Variable X N Sample p 95% CI Z-Value P-Value

(Q) 11 26 0.423077 (0.233175, 0.612979) -0.78 0.433

Using the normal approximation.

Test and CI for One Proportion: (R)

Test of p = 0.5 vs p ≠ 0.5

Event = 2

Variable X N Sample p 95% CI Z-Value P-Value

(R) 7 16 0.437500 (0.194426, 0.680574) -0.50 0.617

Using the normal approximation.

Test and CI for One Proportion: (S)

Test of p = 0.5 vs p ≠ 0.5

Event = 2

Exact

Variable X N Sample p 95% CI P-Value

(S) 6 10 0.600000 (0.262378, 0.878448) 0.754

Test and CI for One Proportion: (T)

Test of p = 0.5 vs p ≠ 0.5

Event = 2

Variable X N Sample p 95% CI Z-Value P-Value

(T) 10 17 0.588235 (0.354285, 0.822186) 0.73 0.467

Using the normal approximation.

Test and CI for One Proportion: (U)

Test of p = 0.5 vs p ≠ 0.5

Event = 1

Exact

Variable X N Sample p 95% Lower Bound P-Value

(U) 6 6 1.000000 0.606962 0.031

Test and CI for One Proportion: (V)

Test of p = 0.5 vs p ≠ 0.5

Event = 2

Variable X N Sample p 95% CI Z-Value P-Value

(V) 7 13 0.538462 (0.267469, 0.809454) 0.28 0.782 Using the normal approximation.

Data column; C82

Test and CI for One Proportion: (W)

Test of p = 0.5 vs p ≠ 0.5

Event = 2

Exact

Variable X N Sample p 95% CI P-Value

(W) 9 11 0.818182 (0.482244, 0.977169) 0.065

Test and CI for One Proportion: (X)

Test of p = 0.5 vs p ≠ 0.5

Event = 2

Variable X N Sample p 95% CI Z-Value P-Value

(X) 10 17 0.588235 (0.354285, 0.822186) 0.73 0.467

Using the normal approximation.

Test and CI for One Proportion: (Y)

Test of p = 0.5 vs p ≠ 0.5

Event = 2

Exact

Variable X N Sample p 95% CI P-Value

(Y) 10 14 0.714286 (0.418965, 0.916111) 0.180

Test and CI for One Proportion: (Z)

Test of p = 0.5 vs p ≠ 0.5

Event = 2

Exact

Variable X N Sample p 95% CI P-Value

(Z) 5 6 0.833333 (0.358765, 0.995789) 0.219

Test and CI for One Proportion: (AA)

Test of p = 0.5 vs p ≠ 0.5

Event = 2

Variable X N Sample p 95% CI Z-Value P-Value

(AA) 8 14 0.571429 (0.312204, 0.830653) 0.53 0.593

Using the normal approximation.

Test and CI for One Proportion: (AB)

Test of p = 0.5 vs p ≠ 0.5

Event = 2

Exact

Variable X N Sample p 95% CI P-Value

(AB) 3 9 0.333333 (0.074855, 0.700705) 0.508

Test and CI for One Proportion: (AC)

Test of p = 0.5 vs p ≠ 0.5

Event = 1

Exact

Variable X N Sample p 95% Lower Bound P-Value

(AC) 8 8 1.000000 0.687656 0.008

Test and CI for One Proportion: (AD)

Test of p = 0.5 vs p ≠ 0.5

Event = 2

Exact

Variable X N Sample p 95% CI P-Value

(AD) 1 7 0.142857 (0.003610, 0.578723) 0.125

Test and CI for One Proportion: (AE)

Test of p = 0.5 vs p ≠ 0.5

Event = 2

Exact

Variable X N Sample p 95% CI P-Value

(AE) 7 9 0.777778 (0.399906, 0.971855) 0.180

Test and CI for One Proportion: (AF)

Test of p = 0.5 vs p ≠ 0.5

Event = 2

Exact

Variable X N Sample p 95% CI P-Value

(AF) 2 10 0.200000 (0.025211, 0.556095) 0.109

Test and CI for One Proportion: (AG)

Test of p = 0.5 vs p ≠ 0.5

Event = 2

Exact

Variable X N Sample p 95% CI P-Value

(AG) 5 8 0.625000 (0.244863, 0.914767) 0.727

Test and CI for One Proportion: (AH)

Test of p = 0.5 vs p ≠ 0.5

Event = 2

Variable X N Sample p 95% CI Z-Value P-Value

(AH) 9 19 0.473684 (0.249173, 0.698196) -0.23 0.819

Using the normal approximation.

Test and CI for One Proportion: (AI)

Test of p = 0.5 vs p ≠ 0.5

Event = 2

Variable X N Sample p 95% CI Z-Value P-Value

(AI) 7 12 0.583333 (0.304394, 0.862273) 0.58 0.564

Using the normal approximation.

Test and CI for One Proportion: (AJ)

Test of p = 0.5 vs p ≠ 0.5

Event = 2

Variable X N Sample p 95% CI Z-Value P-Value

(AJ) 5 12 0.416667 (0.137727, 0.695606) -0.58 0.564

Using the normal approximation.

Test and CI for One Proportion: (AK)

Test of p = 0.5 vs p ≠ 0.5

Event = 2

Exact

Variable X N Sample p 95% CI P-Value

(AK) 1 6 0.166667 (0.004211, 0.641235) 0.219

Test and CI for One Proportion: (AL)

Test of p = 0.5 vs p ≠ 0.5

Event = 2

Exact

Variable X N Sample p 95% CI P-Value

(AL) 5 7 0.714286 (0.290421, 0.963307) 0.453

Test and CI for One Proportion: (AM)

Test of p = 0.5 vs p ≠ 0.5

Event = 2

Exact

Variable X N Sample p 95% CI P-Value

(AM) 5 9 0.555556 (0.212009, 0.863004) 1.000

Test and CI for One Proportion: (AN)

Test of p = 0.5 vs p ≠ 0.5

Event = 2

Exact

Variable X N Sample p 95% CI P-Value

(AN) 4 5 0.800000 (0.283582, 0.994949) 0.375

Test and CI for One Proportion: (AO)

Test of p = 0.5 vs p ≠ 0.5

Event = 2

Exact

Variable X N Sample p 95% CI P-Value

(AO) 5 7 0.714286 (0.290421, 0.963307) 0.453

Test and CI for One Proportion: (AP)

Test of p = 0.5 vs p ≠ 0.5

Event = 2

Variable X N Sample p 95% CI Z-Value P-Value

(AP) 12 47 0.255319 (0.130659, 0.379979) -3.35 0.001

Using the normal approximation.

Test and CI for One Proportion: (AQ)

Test of p = 0.5 vs p ≠ 0.5

Event = 2

Variable X N Sample p 95% CI Z-Value P-Value

(AQ) 7 21 0.333333 (0.131714, 0.534953) -1.53 0.127

Using the normal approximation.

Test and CI for One Proportion: (AR)

Test of p = 0.5 vs p ≠ 0.5

Event = 2 Exact

Variable X N Sample p 95% CI P-Value

(AR) 7 9 0.777778 (0.399906, 0.971855) 0.180

21) WELCH’S T TEST

A) Two-Sample T-Test and CI: LEFT SIGNIFICANTLY LATERALIZED VS RIGHT SIGNIFICANTLY LATERALIZED (ALL HORSES)

Two-sample T for LEFT sig vs RIGHT sig

N Mean StDev SE Mean

LEFT sig 8 71.90 25.2 8.9

RIGHT sig 2 49.21 8.98 6.3

Difference = μ (LEFT sig) - μ (RIGHT sig)

Estimate for difference: 22.7

95% CI for difference: (-20.6, 66.1)

T-Test of difference = 0 (vs ≠): T-Value = 1.21 P-Value = 0.261 DF = 8

Both use Pooled StDev = 23.7660

B) Two-Sample T-Test and CI: ALL HORSES LEFT VS ALL HORSES RIGHT LEG PREFERENT

Two-sample T for (LI)ALL HORS_2 LEFT vs (LI)ALL HORS_1 RIGHT

N Mean StDev SE Mean

(LI)ALL HORS_2 LEFT 21 52.1 28.9 6.3

(LI)ALL HORS_1 RIGHT 22 35.1 18.8 4.0

Difference = μ ((LI)ALL HORS_2 LEFT) - μ ((LI)ALL HORS_1 RIGHT)

Estimate for difference: 17.06

95% CI for difference: (1.85, 32.26)

T-Test of difference = 0 (vs ≠): T-Value = 2.28 P-Value = 0.029 DF = 34

C) Two-Sample T-Test and CI: MARES LEFT VS MARES RIGHT LEG PREFERENT

N Mean StDev SE Mean

LI MARES_L 10 56.1 31.3 9.9

LI MARES_R 11 29.4 18.6 5.6

Difference = μ (LI MARES_L) - μ (LI MARES_R)

Estimate for difference: 26.7

95% CI for difference: (3.4, 50.0)

T-Test of difference = 0 (vs ≠): T-Value = 2.40 P-Value = 0.027 DF = 19

Both use Pooled StDev = 25.4374

D) Two-Sample T-Test and CI: LI GELDINGS LEFT VS GELDINGS RIGHT LEG PREFERENT

Two-sample T for LI GELDINGS_L vs LI GELDINGS_R

N Mean StDev SE Mean

LI GELDINGS_L 6 60.3 25.8 11

LI GELDINGS_R 5 43.7 17.2 7.7

Difference = μ (LI GELDINGS_L) - μ (LI GELDINGS_R)

Estimate for difference: 16.7

95% CI for difference: (-14.0, 47.3)

T-Test of difference = 0 (vs ≠): T-Value = 1.23 P-Value = 0.249 DF = 9

Both use Pooled StDev = 22.3752

E) Two-Sample T-Test and CI: STALLIONS LEFT VS STALLIONS RIGHT LEG PREFERENT

Two-sample T for LI STALLIONS L vs LI STALLIONS R

N Mean StDev SE Mean

LI STALLIONS L 5 34.2 24.6 11

LI STALLIONS R 6 38.2 20.1 8.2

Difference = μ (LI STALLIONS L) - μ (LI STALLIONS R)

Estimate for difference: -4.0

95% CI for difference: (-34.4, 26.4)

T-Test of difference = 0 (vs ≠): T-Value = -0.30 P-Value = 0.773 DF = 9

Both use Pooled StDev = 22.1963

F) Two-Sample T-Test and CI: MALE LEFT VS MALE RIGHT LEG PREFEERENT

Two-sample T for MALE_L vs MALE R

N Mean StDev SE Mean

MALE_L 11 48.4 27.6 8.3

MALE R 11 40.7 18.1 5.5

Difference = μ (MALE_L) - μ (MALE R)

Estimate for difference: 7.78

95% lower bound for difference: -9.38

T-Test of difference = 0 (vs >): T-Value = 0.78 P-Value = 0.222 DF = 20

Both use Pooled StDev = 23.3327

G) Two-Sample T-Test and CI: FEMALE LEFT VS MALE LEFT LEG PREFERENT

Two-sample T for LI MARES_L vs MALE_L

N Mean StDev SE Mean

LI MARES_L 10 56.1 31.3 9.9

MALE_L 11 48.4 27.6 8.3

Difference = μ (LI MARES_L) - μ (MALE_L)

Estimate for difference: 7.7

95% CI for difference: (-19.2, 34.6)

T-Test of difference = 0 (vs ≠): T-Value = 0.60 P-Value = 0.556 DF = 19

Both use Pooled StDev = 29.4086

H) Two-Sample T-Test and CI: FEMALE RIGHT VS MALE RIGHT LEG PREFERENT

Two-sample T for LI MARES_R vs MALE R

N Mean StDev SE Mean

LI MARES_R 11 29.4 18.6 5.6

MALE R 11 40.7 18.1 5.5

Difference = μ (LI MARES_R) - μ (MALE R)

Estimate for difference: -11.22

95% CI for difference: (-27.56, 5.12)

T-Test of difference = 0 (vs ≠): T-Value = -1.43 P-Value = 0.167 DF = 20

Both use Pooled StDev = 18.3712

**22) FURTHER TEST OF LATERALITY DUE TO SEX OF THE HORSE USING LI DATA**

1. Regression Analysis: LI OF ALL HORSES versus MARES, STALLIONS AND GELDINGS

Method

Categorical predictor coding (1, 0)

Analysis of Variance

Source DF Adj SS Adj MS F-Value P-Value

Regression 2 2379 1189 0.47 0.628

MARES STALLIONS GELDINGS 2 2379 1189 0.47 0.628

Error 41 103491 2524

Total 43 105870

Model Summary

S R-sq R-sq(adj) R-sq(pred)

50.2411 2.25% 0.00% 0.00%

Coefficients

Term Coef SE Coef T-Value P-Value VIF

Constant -13.1 15.1 -0.86 0.393

MARES STALLIONS GELDINGS

MARE 2.3 18.6 0.12 0.903 1.50

STALLION 18.4 21.4 0.86 0.396 1.50

Regression Equation

LI ALL HORSES = -13.1 + 0.0 MARES STALLIONS GELDINGS_GELDING

+ 2.3 MARES STALLIONS GELDINGS_MARE + 18.4 MARES STALLIONS GELDINGS_STALLION

1. Regression Analysis: LI ALL HORSES versus MALE AND FEMALE HORSES USING LI DATA

Method

Categorical predictor coding (1, 0)

Analysis of Variance

Source DF Adj SS Adj MS F-Value P-Value

Regression 1 525 525.4 0.21 0.650

MALE AND FEMALE 1 525 525.4 0.21 0.650

Error 42 105345 2508.2

Total 43 105870

Model Summary

S R-sq R-sq(adj) R-sq(pred)

50.0820 0.50% 0.00% 0.00%

Coefficients

Term Coef SE Coef T-Value P-Value VIF

Constant -10.8 10.7 -1.01 0.318

MALE AND FEMALE

MALE 6.9 15.1 0.46 0.650 1.00

Regression Equation

LI ALL HORSES = -10.8 + 0.0 MALE AND FEMALE_FEMALE + 6.9 MALE AND FEMALE_MALE

**23) CRONBACH'S ALPHA TEST**

Item Analysis of OBSERVER1, OBSERVER2, OBSERVER3, OBSERVER4 (REPRODUCEABILITY-CRONCACH'S ALPHA)

Correlation Matrix

OBSERVER1 OBSERVER2 OBSERVER3

OBSERVER2 0.801

OBSERVER3 0.721 0.600

OBSERVER4 0.801 0.680 0.520

Cell Contents: Pearson correlation

Item and Total Statistics

Total

Variable Count Mean StDev

OBSERVER1 50 1.4800 0.5047

OBSERVER2 50 1.5000 0.5051

OBSERVER3 50 1.5000 0.5051

OBSERVER4 50 1.5000 0.5051

Total 50 5.9800 1.7669

Cronbach’s alpha = 0.8977

Omitted Item Statistics

Adj. Adj. Squared

Omitted Total Total Item-Adj. Multiple Cronbach’s

Variable Mean StDev Total Corr Corr Alpha

OBSERVER1 4.500 1.298 0.9038 0.8197 0.8182

OBSERVER2 4.480 1.344 0.7818 0.6471 0.8646

OBSERVER3 4.480 1.389 0.6693 0.5307 0.9050

OBSERVER4 4.480 1.359 0.7434 0.6526 0.8786
